# DNA Polymerase κ Is a Key Cellular Factor for the Formation of Covalently Closed Circular DNA of Hepatitis B Virus

**DOI:** 10.1371/journal.ppat.1005893

**Published:** 2016-10-26

**Authors:** Yonghe Qi, Zhenchao Gao, Guangwei Xu, Bo Peng, Chenxuan Liu, Huan Yan, Qiyan Yao, Guoliang Sun, Yang Liu, Dingbin Tang, Zilin Song, Wenhui He, Yinyan Sun, Ju-Tao Guo, Wenhui Li

**Affiliations:** 1 National Institute of Biological Sciences, Beijing, China; 2 Graduate program in School of Life Sciences, Peking University, Beijing, China; 3 College of Life Sciences Beijing Normal University, Beijing, China; 4 School of Life Science, Tsinghua University, Beijing, China; 5 Baruch S. Blumberg Institute, Doylestown, Pennsylvania, United States of America; The Pennsylvania State University College of Medicine, UNITED STATES

## Abstract

Hepatitis B virus (HBV) infection of hepatocytes begins by binding to its cellular receptor sodium taurocholate cotransporting polypeptide (NTCP), followed by the internalization of viral nucleocapsid into the cytoplasm. The viral relaxed circular (rc) DNA genome in nucleocapsid is transported into the nucleus and converted into covalently closed circular (ccc) DNA to serve as a viral persistence reservoir that is refractory to current antiviral therapies. Host DNA repair enzymes have been speculated to catalyze the conversion of rcDNA to cccDNA, however, the DNA polymerase(s) that fills the gap in the plus strand of rcDNA remains to be determined. Here we conducted targeted genetic screening in combination with chemical inhibition to identify the cellular DNA polymerase(s) responsible for cccDNA formation, and exploited recombinant HBV with capsid coding deficiency which infects HepG2-NTCP cells with similar efficiency of wild-type HBV to assure cccDNA synthesis is exclusively from *de novo* HBV infection. We found that DNA polymerase κ (POLK), a Y-family DNA polymerase with maximum activity in non-dividing cells, substantially contributes to cccDNA formation during *de novo* HBV infection. Depleting gene expression of POLK in HepG2-NTCP cells by either siRNA knockdown or CRISPR/Cas9 knockout inhibited the conversion of rcDNA into cccDNA, while the diminished cccDNA formation in, and hence the viral infection of, the knockout cells could be effectively rescued by ectopic expression of POLK. These studies revealed that POLK is a crucial host factor required for cccDNA formation during a *de novo* HBV infection and suggest that POLK may be a potential target for developing antivirals against HBV.

## Author Summary

HBV chronically infects 240 million people worldwide. Persistent HBV infection relies on stable maintenance of the nuclear form of viral genome, the covalently closed circular (ccc) DNA. However, the molecular mechanism underlying the conversion of HBV genomic relaxed circular (rc) DNA into cccDNA remains elusive. Our studies reported herein provide unambiguous evidence suggesting that host DNA polymerase κ (POLK) is required for repairing the gap of rcDNA and formation of cccDNA in a *de novo* HBV infection. POLK is thus a potential therapeutic target for treatment of chronic hepatitis B.

## Introduction

Despite the availability of effective vaccines for more than three decades, hepatitis B virus (HBV) still persistently infects 240 million people worldwide [1, 2]. Antiviral therapies with nucleos(t)ide analog inhibitors of HBV reverse transcriptase dramatically reduce the viral load, significantly improve the liver function and lower the incidence of liver failure and hepatocellular carcinoma, but fail to cure the viral infection [3, 4], due to the persistence of covalently closed circular (ccc) DNA in the nuclei of infected hepatocytes [5–8]. Hence, better understanding the molecular mechanisms underlying the formation and maintenance of cccDNA is critical for development of novel therapeutics to cure chronic HBV infection.

HBV is the prototype member of *Hepadnaviridae* family and contains a relaxed circular (rc) partially double-stranded DNA genome with its DNA polymerase covalently linked to the 5’ terminus of minus strand [9]. While the minus strand of the rcDNA is completely synthesized with a redundant overhang at the 5’ end, the plus strand is incompletely synthesized, leaving a 3’ terminal gap of variable length [9]. HBV replicates its DNA genome *via* reverse transcription of an RNA intermediate, the pregenomic (pg) RNA [10]. Briefly, HBV entry into hepatocytes is mediated by its host cellular receptor human sodium taurocholate cotransporting polypeptide (NTCP) [11–14]. Upon entry into the cytoplasm of hepatocytes, rcDNA in the nucleocapsid is transported into the nucleus and converted into an episomal cccDNA, which is assembled into a minichromosome to serve as the template for the transcription of viral mRNAs [15, 16]. Following the synthesis of viral proteins, viral DNA polymerase binds to a stem-loop structure (termed epsilon) within the 5’ region of pgRNA to initiate its packaging into nucleocapsids where the pgRNA is reverse transcribed to progeny rcDNA [17]. The progeny “mature” rcDNA-containing nucleocapsids can be either enveloped and secreted out of the cell as virion particles or might be redirected into the nucleus to amplify the cccDNA pool [18–20] [21]. Thus, the formation and intracellular amplification of cccDNA plays a central role in the establishment and maintenance of persistent infection. Biochemically, conversion of rcDNA to cccDNA requires the removal of the viral DNA polymerase and RNA primer from the 5’-terminus of minus strand and plus strand DNA, respectively; filling in the gap in plus strand DNA, trimming and ligating the ends of both strands. Although it is speculated that all those reactions are most probably catalyzed by host cellular DNA repair enzymes, identification of the cellular proteins responsible for cccDNA formation has thus far only achieved limited success. For instance, tyrosyl-DNA phosphodiesterase-2 (Tdp2), a cellular enzyme responsible for cleavage of tyrosyl-5' DNA linkages formed between topoisomerase II and cellular DNA [22], can release covalently linked RT from the 5’ end of minus-strand DNA *in vitro* [23, 24], and has recently been shown to cleave the tyrosyl-minus strand DNA linkage of HBV. However, Tdp2 gene knockout only slows down the formation of duck hepatitis B virus (DHBV) cccDNA from intracellular amplification pathway, but does not inhibit HBV cccDNA formation in HBV infection of HepG2-NTCP cells [25, 26]. In addition to rcDNA, cccDNA can also be formed from double stranded linear DNA (dslDNA) [27], which is derived from *in situ* priming of plus strand DNA synthesis [28]. Interestingly, Ku80, a component of non-homologous end joining DNA repair pathway, has been reported to play an essential role in the synthesis of DHBV cccDNA from dslDNA, but not rcDNA [29].

Completion of plus strand DNA synthesis, or “filling the gap” in the plus strand of rcDNA, is essential for cccDNA synthesis. Studies of DHBV and woodchuck hepatitis virus (WHV), two hepadnaviruses distinct from human HBV but readily cultivable *in vitro*, showed that viral DNA polymerase inhibitors did not prevent cccDNA formation in the infection of primary hepatocytes of ducks and woodchucks, implying that viral DNA polymerase may be dispensable, while cellular DNA polymerase activity is required for the completion of plus strand DNA synthesis [30–33]. Moreover, continued treatment of primary tupaia hepatocytes and HepaRG cells with viral polymerase inhibitors during HBV infection did not inhibit HBV cccDNA formation [34, 35], further suggesting that cellular factor(s) play an important role. Here we set out to identify the DNA polymerase(s) that complete(s) the plus strand DNA synthesis required for HBV cccDNA formation. In HepG2-NTCP cells infected with HBV that is deficient in core protein production, we unambiguously demonstrated that viral DNA polymerase activity is not required for cccDNA formation in a *de novo* infection. Instead, a focused RNA interference loss-of-function screening identified POLK as a crucial cellular polymerase supporting HBV infection. Both knockdown and knockout of POLK impaired the conversion of rcDNA to cccDNA, which could be rescued by ectopic expression of POLK. Our findings thus suggest that POLK is a key host factor required for cccDNA formation during a *de novo* HBV infection, and therefore, a potential target for therapeutic intervention of chronic hepatitis B.

## Results

### Kinetic of cccDNA formation in HepG2-NTCP cells infected by HBV

**Fig 1:**
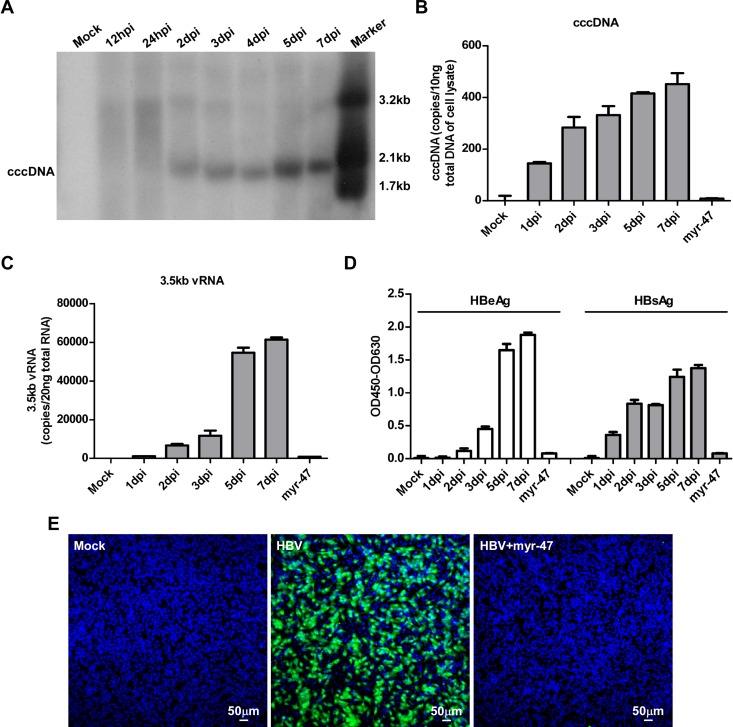
Kinetics of cccDNA formation in HepG2-NTCP cells infected by HBV. HepG2-NTCP cells were inoculated for 24 h with HBV in the presence of 4% PEG8000 with or without 100 nM preS1-myr-47 lipopeptide. The cells were then cultured for 7 days in PMM. **(A)** HBV cccDNA was extracted at indicated times post infection using Hirt method and followed by digestion with *Hind*III. The DNA samples were analyzed by Southern blot with an [α-^32^P]dCTP-labeled full-length HBV DNA probe. 100 pg each of 3.2kb, 2.1kb and 1.7kb HBV DNA fragments were used as molecular weight markers; dpi: days post infection; cccDNA: covalently closed circular DNA. **(B-D)** Total cellular DNA and RNA were extracted from HBV infected HepG2-NTCP cells. HBV cccDNA (B) and 3.5kb vRNA (C) levels at the indicated times post-infection were quantified by real-time qPCR assays. Secreted HBeAg and HBsAg were measured by ELISA (D). Representatives of three independent experiments are presented. **(E)** On 7 dpi, the cells were fixed and stained for intracellular HBcAg with mcAb 1C10 (green) and nuclei were stained with DAPI (blue). Images were captured with a Nikon A1-R confocal microscopy. Scale bars, 50 μm.

To investigate the molecular mechanism of HBV cccDNA formation, we first determined the kinetics of cccDNA synthesis in wild-type HBV infected HepG2-NTCP cells. Using Southern blot analysis, we examined HBV cccDNA in Hirt extracts of infected cells at various time points post infection. The identity of the cccDNA, which migrated at the position of 2.1kb linear DNA, was confirmed by the band shift to a 3.2kb DNA species corresponding to the size of unit-length linear HBV genomic DNA upon digestion by *EcoR*I, but not *Hind*III (S1 Fig). As shown in Fig 1A, the protein-free rcDNA species accumulated at 12 and 24 h post infection and cccDNA became detectable at day 2 post-infection, followed by a modest increase in the next 5 days. The appearance of cccDNA was coincident with reduction of the protein-free rcDNA in the infected cells. Consistently, based on the quantitative analysis of cccDNA using a more sensitive real-time PCR assay, cccDNA was detectable at 24 h post-infection, markedly increased in the first 2 days post-infection, followed by a slower increase to approximately 3 copies per infected cell in the next 5 days (Fig 1B). Similar to the kinetics of cccDNA synthesis, the levels of intracellular HBV 3.5kb vRNA (Fig 1C) as well as secreted HBeAg and HBsAg (Fig 1D) gradually increased following HBV infection. Immunostaining revealed that over 60% of HepG2-NTCP cells were HBcAg positive at day 7 post infection (Fig 1E). As expected, HBV preS1 myr-47 lipopeptide (myr-47) completely blocked the viral infection. Together, these results demonstrated that HepG2-NTCP cells support an efficient HBV infection, resulting in readily detectable cccDNA formation, gene expression and secretion of viral proteins. Hence, the HBV infection cell culture system is suitable for identification of viral and host cellular factors required for cccDNA formation during a *de novo* HBV infection.

### HBV DNA polymerase activity is dispensable for cccDNA formation

**Fig 2:**
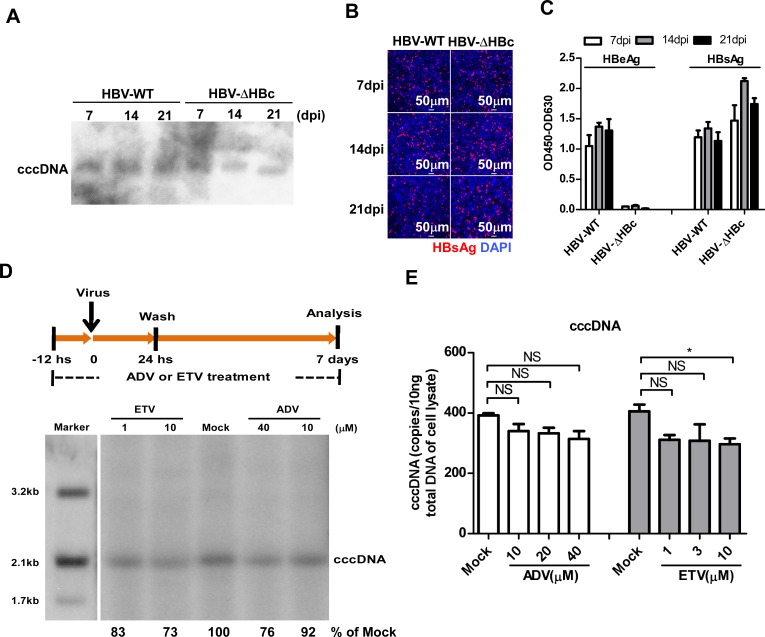
HBV DNA polymerase activity is dispensable for cccDNA biosynthesis in a *de novo* infection. **(A-C)** HepG2-NTCP cells were infected with a multiplicity of 100 genome equivalents (mge) of wild-type or HBcAg-deficient (HBV-ΔHBc) virus. On the indicated time points after infection, HBV cccDNA was determined by Southern blot analysis (A). Intracellular HBsAg was stained with mcAb 17B9 (red) and nuclei were stained with DAPI (blue). Images were taken with a Nikon A1-R confocal microscopy. Scale bars, 50 μm (B). Secreted HBeAg and HBsAg were measured by ELISA (C). Data are representatives of three independent experiments. **(D-E)** HepG2-NTCP cells were mock-treated or treated with the indicated concentrations of adefovir (ADV) or entecavir (ETV) from 12 h before HBV-ΔHBc infection to 7 dpi as depicted in the top panel (D). HBV cccDNA in the infected cells were detected by Southern blot analysis (D). The intensity of HBV cccDNA bands were determined by Image J and the relative amounts of cccDNA were expressed as the percentage of that in the mock-treated control cells. HBV cccDNA was also quantified by a qPCR assay (E). Data are representatives of three independent experiments. Data were analyzed by an unpaired two-tailed *t* test. NS: non-significant; * p<0.05.

As stated above, nuclear HBV cccDNA pool is established by direct conversion of rcDNA from incoming virions and supposedly *via* intracellular amplification pathway from rcDNA in the cytoplasmic progeny nucleocapsids. It is clear that viral DNA polymerase activity is essential for the intracellular amplification of cccDNA, but its role in cccDNA formation from incoming virions, specifically, in filling the gap in the plus strand of rcDNA, remains to be rigorously examined. Providing an unambiguous answer to this question is challenging due to the apparently indistinguishable nature of cccDNA synthesized from the two different pathways. In order to thoroughly determine the role of HBV DNA polymerase in cccDNA formation during a *de nove* infection, we first produced HBV virion particles containing genomic DNA with a stop codon at the 38^th^ codon (Y) of the core gene open reading frame, designated as HBV-ΔHBc. As depicted in S2 Fig, this was achieved by co-transfection of Huh-7 cells with plasmid containing 1.05-mer HBV DNA with the desired mutation and plasmid expressing HBV core protein. HBV-ΔHBc virions were harvested from the culture fluid and purified by ultracentrifugation. Due to its inability to produce capsid protein, HBV-ΔHBc infection of cells will not be able to support progeny viral DNA synthesis and formation of cccDNA through the intracellular amplification pathway. Hence, HBV-ΔHBc infection provides a unique opportunity to investigate the role of DNA polymerases in cccDNA formation from incoming virions. HepG2-NTCP cells were infected with a multiplicity of 100 genome equivalents (mge) of wild-type HBV and HBV-ΔHBc virions, respectively. Notably, HBV-ΔHBc successfully infected HepG2-NTCP cells and formed cccDNA at a level comparable to that of wild-type HBV on day 7 post infection and persisted during the following two weeks of extended culturing (Fig 2A). Similarly, immunostaining analysis showed that the levels of intracellular HBsAg were similar in both wild-type HBV and HBV-ΔHBc infected cells during the prolonged 21 days of culture (Fig 2B). As expected, HBV-ΔHBc infected cells did not produce HBeAg in the culture supernatant due to the stop codon mutation. Meanwhile, HBV-ΔHBc infected cells secreted a slightly higher level of HBsAg than wild-type HBV infected cells at the indicated time points post infection (Fig 2C). Together, the results indicate that cccDNA in HBV infected HepG2-NTCP cells were mainly synthesized from input viral rcDNA and that the intracellular amplification pathway did not significantly contribute to the establishment of cccDNA pool under this experimental condition.

To directly examine whether HBV polymerase activity is required for conversion of viral rcDNA from the input viruses to cccDNA, HepG2-NTCP cells were infected with HBV-ΔHBc in the presence or absence of adefovir (ADV) or entecavir (ETV). As shown in Fig 2D and 2E, neither of the compounds prevented cccDNA accumulation in HBV-ΔHBc infected cells. Quantitative analysis indicated that the viral DNA polymerase inhibitor treatment at relatively high concentration reduced cccDNA accumulation by less than 27%. Interestingly, similar ADV and ETV treatment of wild-type HBV infected cells reduced cccDNA accumulation by 28 to 42% (S3 Fig). These results indicate that the activity of HBV DNA polymerase is dispensable in cccDNA formation from incoming virion rcDNA, and re-enforce the notion that intracellular amplification of cccDNA does not play an important role in establishment of cccDNA pool in the HBV-infected hepatoma cells.

### Focused siRNA screening identifies cellular DNA polymerases involving in HBV infection

**Fig 3:**
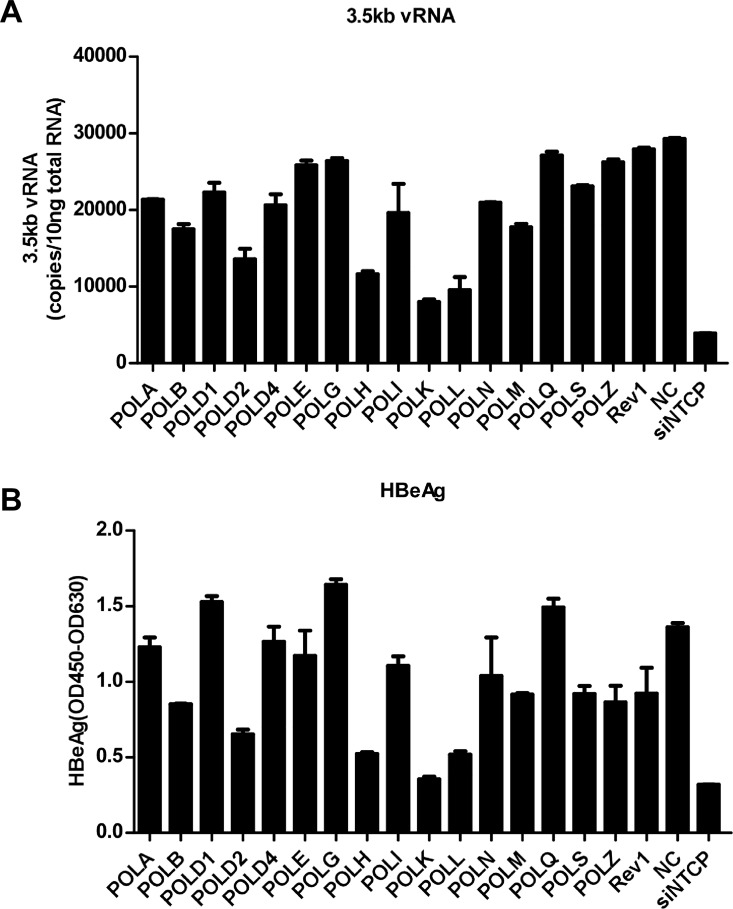
Focused siRNA screening of host DNA polymerases involved in HBV infection. **(A and B)** siRNA targeting different cellular DNA polymerase was transfected into HepG2-NTCP cells with Lipofectamine RNAiMAX. Three days after transfection, cells were challenged with HBV. HBV infection was assessed by measuring intracellular 3.5kb vRNA with a qPCR assay on 7 dpi (A) as well as secreted HBeAg (B) at the indicated days post-infection with ELISA. siPOLH-1, siPOLK-1 and siPOLL-1 listed in S1 Table were used in this RNAi screen, NTCP-specific siRNA served as a positive control for inhibition of HBV infection, and NC siRNA served as a negative control. The results are presented as mean ± standard deviation (SD).

**Fig 4:**
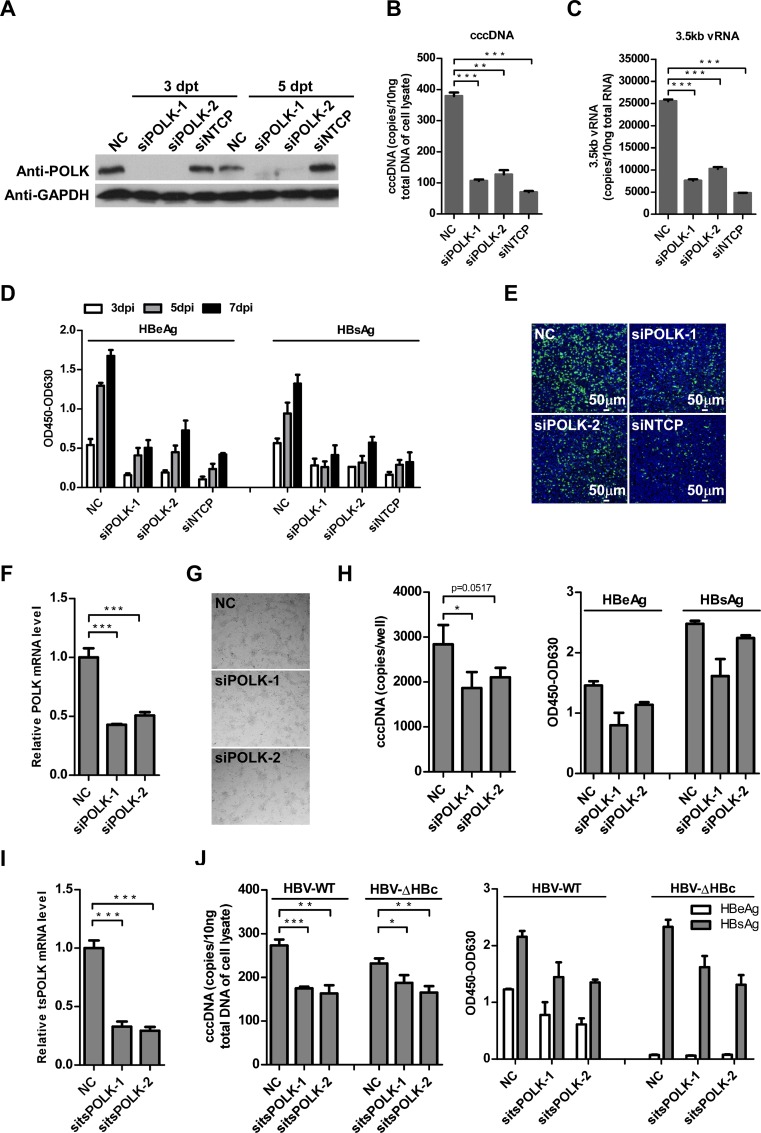
Silencing of POLK impairs *de novo* HBV infection. **(A)** HepG2-NTCP cells were transfected with siRNA targeting POLK, NTCP, or a negative control siRNA (NC). On three and five days post-transfection (dpt), cells were lysed with RIPA buffer supplemented with a protease inhibitor cocktail, respectively. Equal amounts of total cell lysates were subjected to Western blot analysis with antibodies against POLK or GAPDH. **(B-E)** HepG2-NTCP cells were transfected with siRNA targeting POLK or NTCP and infected with HBV at 3 days post siRNA transfection. HBV cccDNA (B) and 3.5kb vRNA (C) levels on 7 dpi were quantified by qPCR assays. Secreted HBeAg and HBsAg at 3, 5 and 7 dpi were measured by ELISA (D). Intracellular HBcAg expression on 7 dpi was detected by immunostaining (E). HBcAg was stained with mcAb 1C10 (green). Nuclei were stained with DAPI (blue). siNTCP was used as a positive control, siRNA NC served as a negative control. Images were captured using a Nikon A1-R confocal microscopy. Scale bars, 50 μm. Data are representatives of three independent experiments. Data were analyzed by an unpaired two-tailed *t* test. ** p<0.01 and *** p<0.001. **(F and G)** HepaRG cells were transfected with siRNA targeting POLK, or a negative control (NC), three days later, total RNA was extracted and reverse-transcribed into cDNA, the mRNA level of POLK was determined by qRT-PCR and normalized to GAPDH expression, respectively. The relative expression of POLK in NC treated sample is set at 1.0. Mean and SD are presented from three independent measurements. Data were analyzed by an unpaired two-tailed *t* test, *** p<0.001 (F). The cytotoxicity of siRNA on HepaRG cells was investigated by fluorescence microscope on 5 days post siRNA transfection (G). **(H)** HepaRG cells were transfected with siRNA targeting POLK or a negative control (NC) and infected with HBV at 3 days post siRNA transfection. On 7 days post infection, HBV cccDNA (left) levels were quantified by qPCR assays. Secreted HBeAg and HBsAg were measured by ELISA (right). Three independent measurements were performed. Data were analyzed by an unpaired two-tailed *t* test, * p<0.05. **(I)** Primary tupaia hepatocytes were transfected with siRNA targeting tsPOLK, or a negative control siRNA (sitsNC). Forty-eight hours later, total RNA was extracted and reverse-transcribed into cDNA, the mRNA level of tsPOLK was determined by qRT-PCR and normalized to tsGAPDH expression, respectively. Three independent measurements were conducted. Data were analyzed by an unpaired two-tailed *t* test, *** p<0.001. **(J)** siRNA-treated primary tupaia hepatocytes were infected with wild-type (HBV-WT) or HBcAg-deficient (HBV-ΔHBc) virus at 2 days post siRNA transfection. On 7 dpi, HBV cccDNA levels were quantified by qPCR assays (left). Secreted HBeAg and HBsAg were measured by ELISA (right). Three independent measurements were performed. Data were analyzed by an unpaired two-tailed *t* test, * p<0.05, ** p<0.01, *** p<0.001.

There are fifteen different DNA polymerases in mammalian cells. They function in genome replication, DNA repair, and translesion DNA synthesis (TLS) of damaged DNA. To identify which host DNA polymerase contributes to HBV infection, we conducted a focused siRNA screening by targeting cellular DNA polymerase genes. We assessed HBV infection upon silencing the expression of individual DNA polymerase gene in HepG2-NTCP cells. All siRNA sequences targeting cellular DNA polymerase genes were obtained from previous studies; these individual siRNAs could unequivocally discriminate between the mRNAs of the 15 different polymerases [36]. An siRNA targeting NTCP and a scrambled sequence (siRNA NC) were used as positive and negative controls, respectively. The levels of intracellular 3.5kb viral RNA as well as secreted HBeAg in culture supernatants were measured by qRT-PCR and ELISA. As shown in Fig 3, among 15 cellular DNA polymerases, knocking down POLK, POLH or POLL gene expression significantly decreased the levels of intracellular 3.5kb viral RNA as well as secreted HBeAg, POLB and POLD2 silencing only led to a modest decrease of intracellular 3.5kb viral RNA production. Of note, no significant cytotoxic effect was observed upon silencing any of the host DNA polymerases (S4 Fig). To confirm the specificity of the siRNA in the targeted RNAi screen, we evaluated the efficacy of single and combined dual siRNA-mediated knockdown of POLK, POLL and POLH genes (S5 Fig and Fig 4), respectively. Western blot analysis or qRT-PCR assay showed that each of the siRNAs specifically reduced the expression of its targeted DNA polymerase gene (S5A Fig and Fig 4). Among the three cellular polymerases, silencing of POLK exhibited the most dramatic inhibitory effect on HBV infection, with approximately 74% reduction of HBV infection as judged by HBeAg level, which was comparable to the efficiency of silencing NTCP expression that reduced HBV infection by 80% (S5C Fig). Similar extents of reduction were also observed for intracellular 3.5kb vRNA (S5B Fig) and HBcAg levels (S5D Fig) in POLK siRNA transfected cultures. Knock-down of POLL gene also led to a notable decrease of intracellular 3.5kb viral RNA and HBcAg. Interestingly, at the same total concentrations of siRNAs, compared with only knock-down POLK gene (by siRNAs of NC and POLK), dual siRNAs-mediated knockdown of POLK and POLL, or, POLK and POLH demonstrated enhanced inhibition of HBV infection, but did not completely abolish HBV infection (S5E–S5G Fig). These results thus suggest that while POLK, POLL and POLH each individually could contribute to *de novo* HBV infection, POLK clearly plays a critical role.

POLK belongs to the Y family of DNA polymerases, which functions in translesion synthesis and nucleotide excision DNA repair. Its enzymatic activity is resistant to aphidicolin (APH) and dideoxynucleotides [37]. In line with this, APH treatment did not inhibit HBV cccDNA synthesis and secretion of HBeAg in HBV infection of HepG2-NTCP cells (S6 Fig). The results also imply that APH-sensitive DNA polymerases (e.g. POLA, POLE) are not required for HBV infection of HepG2-NTCP cells, which is consistent with the results obtained from siRNA knockdown experiments.

### Silencing of POLK impairs *de novo* HBV infection and ectopic expression of POLK rescues the infection

To confirm the role of POLK in HBV cccDNA formation, two individual siRNAs targeting different region of POLK gene were transiently transfected into HepG2-NTCP cells. The siRNAs significantly reduced the expression levels of endogenous POLK at day 3 and 5 post transfection (Fig 4A). We found that POLK knockdown led to 70% reduction of cccDNA levels compared to that in control siRNA transfected cells on day 7 post infection (Fig 4B). In line with this observation, the 3.5kb vRNA level was also reduced by approximately 60% in POLK knockdown cells (Fig 4C). HBeAg and HBsAg levels in culture supernatants were reduced to less than 30% of that from control siRNA transfected cells (Fig 4D). Immunostaining of intracellular HBcAg also showed significant decrease in POLK knockdown cultures (Fig 4E).

To investigate whether POLK plays a similar role in other *in vitro* HBV infection experimental models, we conducted siRNA-mediated knockdown of POLK in HepaRG cells (Fig 4F–4H) and primary tupaia hepatocytes (PTH) (Fig 4I and 4J), respectively. Compared to a negative control (NC), differentiated HepaRG cells treated with two individual siRNAs targeting POLK reduced the expression level of POLK, decreased HBV cccDNA synthesis and reduced secretion of HBeAg and HBsAg. No cytotoxic effect was observed at 5 days post siRNA transfection (Fig 4G). Consistent with the results for HBV infected HepG2-NTCP and HepaRG cells, silencing of tupaia POLK by siRNA (sitsPOLK) led to a marked decrease of cccDNA synthesis and production of HBeAg and HBsAg on day 7 post infection of PTH. Moreover, similar to infection by wild-type HBV, transfection of sitsPOLK also significantly reduced the levels of intracellular HBV cccDNA and HBsAg in HBV-ΔHBc infected PTHs (Fig 4J). Importantly, siRNA knockdown of POLK did not reduce the infection efficiency of HDV (S7A Fig) or an EGFP-encoding VSV-G pseudotyped lentivirus (VSV-EGFP) (S7B Fig), demonstrating that POLK has a specific role in HBV infection. Together, these data suggest that POLK is required for *de novo* HBV infection and depletion of POLK diminishes HBV cccDNA synthesis.

In order to further confirm that POLK is responsible for formation of HBV cccDNA, we intended to restore POLK expression in the siRNA transfected cells. To achieve this goal, an expression plasmid of POLK fused with GFP at N-terminus (GFP-POLK-wt) was constructed. Silent mutations were introduced to siPOLK1-targeting sequence for expression of the siRNA-resistant POLK mRNA (GFP-POLK-res). HepG2-NTCP cells were transduced with VSV-G protein pseudotyped lentiviruses expressing GFP-POLK-res, GFP-POLK-wt or GFP alone, respectively. The cells were then transfected with siPOLK-1. Fluorescence microscopic analysis showed that POLK localized in the nuclei, and siPOLK-1 dramatically reduced the expression of GFP-POLK-wt, but not GFP-POLK-res, suggesting that the expression of GFP-POLK-res is indeed resistant to siPOLK-1 (S8A Fig). We next performed HBV infection assay. As shown in S8B–S8D Fig, while siPOLK-1 transfection efficiently reduced HBV cccDNA formation as well as 3.5 kb vRNA expression and HBeAg secretion in HepG2-NTCP cells transduced with control lentivirus expressing GFP, the effects of siPOLK-1 transfection on cccDNA formation and function in HepG2-NTCP cells expressing POLK, in particular GFP-POLK-res were significantly attenuated. The results thus suggest that ectopic expression of POLK partially rescued the suppression of HBV cccDNA formation caused by POLK-targeting siRNA.

### POLK substantially contributes to HBV cccDNA synthesis

**Fig 5:**
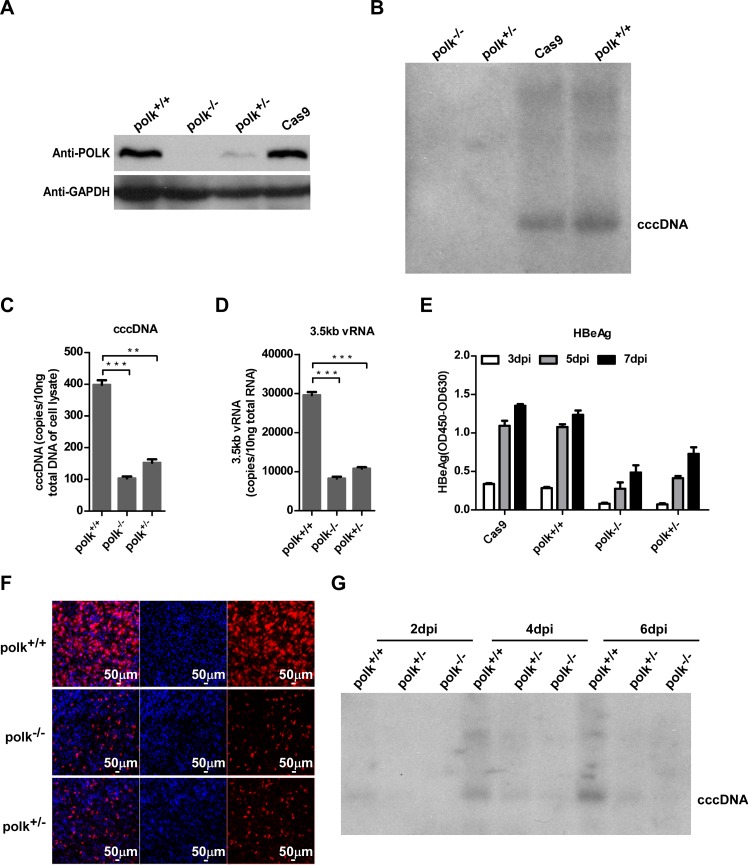
POLK is required for HBV cccDNA synthesis. **(A)** POLK expression in parental HepG2-NTCP cells and HepG2-NTCP cell clones bearing disrupted *polk* gene in one (HepG2-NTCP^*polk+/-*^) or both alleles (HepG2-NTCP^*polk-/-*^) was determined by Western blot assay. GAPDH protein served as a loading control. **(B-F)** The indicated HepG2-NTCP-derived cell lines were infected with HBV. HBV cccDNA on 7 dpi was detected by Southern blot (B). The amounts of cccDNA (C) and 3.5kb vRNA (D) on 7 dpi were measured by qPCR assays. Data were analyzed by an unpaired two-tailed *t* test. ** p<0.01 and *** p<0.001. Secreted HBeAg from the indicated HepG2-NTCP cells on 3, 5, 7 dpi was measured by ELISA (E). HBcAg in the infected cultures was stained with 1C10 mcAb (red). Nuclei were stained with DAPI (blue) (F). Images were captured with a Nikon A1-R confocal microscopy, scale bars, 50 μm. **(G)** The timecourse of cccDNA synthesis in HBV infected HepG2-NTCP cells with intact or disrupted POLK gene was determined by Southern blot analysis.

To rigorously determine the function of POLK in HBV cccDNA synthesis, we took advantage of the CRISPR/Cas9 system to generate POLK knockout in HepG2-NTCP cells (S9 Fig). We first created a stable HepG2-NTCP/Cas9 cell line that constitutively expresses Cas9. The cell line was then infected with lentivirus encoding an EGFP protein and sgRNA targeting exon 2 of *polk* gene. By monitoring the expression of EGFP, we could assess the sgRNA transduction efficiency. On day 3 post-transduction, EGFP positive cells were sorted and expanded by culturing for additional 10 days. Independent HepG2-NTCP clones with successful *polk* gene editing (HepG2-NTCP^*polk+/-*^and HepG2-NTCP^*polk-/-*^) were identified and used for further studies. Sequencing analysis revealed that the clones have a frame shift in the coding region owing to nucleotide deletions, which resulted in the disruption of intact POLK protein expression. Western blotting analysis confirmed that the expression of POLK protein was reduced in HepG2-NTCP^*polk+/-*^ and abolished in HepG2-NTCP^*polk-/-*^ clones (Fig 5A). Knockout POLK in HepG2-NTCP cells did not affect the viability of HepG2-NTCP cells. NTCP level remained unchanged as compared to that in parental HepG2-NTCP cells (S10A Fig). Consistently, a functional assay showed that HepG2-NTCP^*polk-/-*^ cells were able to uptake [^3^H]-labeled taurocholate (S10B Fig) and supported HDV infection (S10C Fig) at an efficiency similar to that of the parental HepG2-NTCP cells.

We next carried out HBV infection assay with these stable cell lines described above. The levels of cccDNA were examined by Southern blotting analysis at day 7 post-infection. No visible band of cccDNA could be detected in HepG2-NTCP^*polk-/-*^ cells, indicating that lack of POLK impaired HBV cccDNA synthesis (Fig 5B). Quantitative PCR also showed that the amounts of cccDNA decreased by approximately 3- or 4-fold in the cells with reduced (HepG2-NTCP^*polk+/-*^) and abolished (HepG2-NTCP^*polk-/-*^) POLK expression, respectively (Fig 5C). ELISA analysis showed that secreted HBeAg and HBsAg levels were markedly decreased from HepG2-NTCP^*polk-/-*^ cells (Fig 5E). Similarly, depletion of POLK also dramatically reduced intracellular HBcAg, which was closely related to intracellular 3.5kb vRNA levels (Fig 5D and 5F). Importantly, a time course analysis using Southern blot assay showed that cccDNA was readily detectable in parental HepG2-NTCP cells at day 2 post infection and modestly increased in the next 4 days. In contrast, cccDNA only became detectable in HepG2-NTCP^*polk+/ -*^cells containing one intact *polk* allele at day 4 and day 6 post infection in much reduced amounts, compared to that in the parental HepG2-NTCP cells at the same time points. Only very faint cccDNA bands could be detected in HepG2-NTCP^*polk-/-*^ cells with both *polk* alleles disrupted during the same time period (Fig 5G). In agreement with the cccDNA formation results, POLK knockout cells produced low level HBeAg and HBsAg.

**Fig 6:**
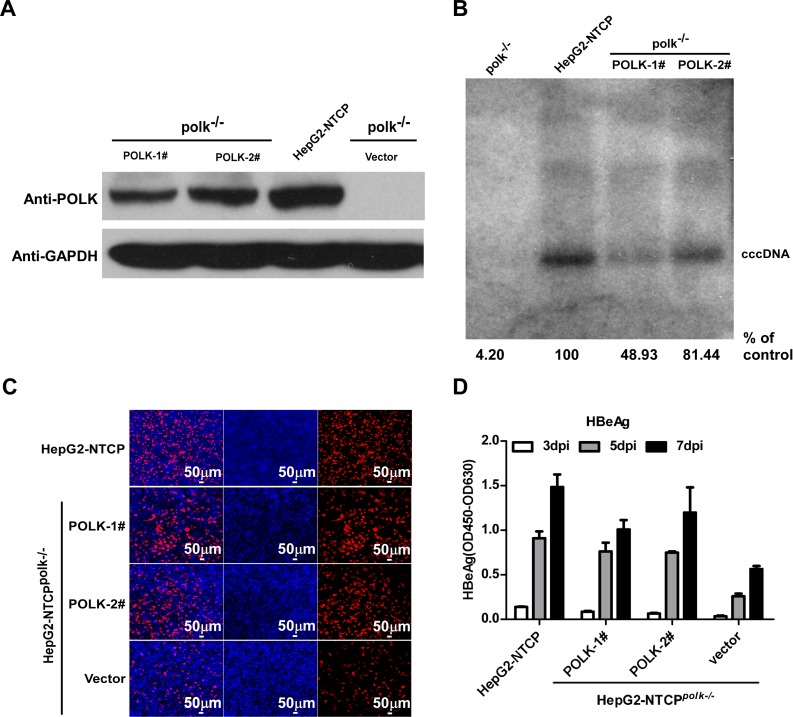
Ectopic expression of POLK rescues HBV infection in POLK deficient HepG2-NTCP cells. **(A)** Expression of POLK in parental HepG2-NTCP and HepG2-NTCP^*polk-/-*^ cells transduced with lentivirus expressing wild-type POLK or empty vector were determined by Western blot analysis. GAPDH served as a loading control. POLK-1# and POLK-2# are two clones expressing exogenous POLK in HepG2-NTCP^*polk-/-*^ cells. **(B-D)** Parental HepG2-NTCP and HepG2-NTCP^*polk-/-*^ cells transduced with lentivirus expressing wild-type POLK or empty vector were infected by HBV. HBV cccDNA in the infected cultures on 7 dpi were detected by Southern blot analysis. The intensity of HBV cccDNA bands were determined by Image J and the relative amounts of cccDNA were expressed as the percentage of that in the infected parental HepG2-NTCP cells (B). HBcAg in the infected cultures on 7 dpi was detected by immunostaining with 1C10 mcAb (red). Cell nuclei were stained with DAPI (blue) (C). Scale bars, 50 μm. Secreted HBeAg from the infected cultures at 3, 5, 7 dpi were determined by ELISA (D).

To confirm the function of POLK in HBV cccDNA synthesis, we restored POLK expression in HepG2-NTCP^*polk-/*-^cells by stable transduction of lentivirus expressing POLK. HepG2-NTCP^*polk-/-*^ cell clones with defective endogenous *polk* but expressing exogenous POLK were established. Production of POLK protein was largely restored in two independent cell clones as demonstrated by Western blot analysis (Fig 6A). The amount of cccDNA upon HBV infection was assessed by Southern blot. Remarkably, HBV infection in these two clones was rescued, and the cccDNA level correlated with the expression levels of POLK in these cells (Fig 6B). In contrast, transduction of HepG2-NTCP^*polk-/-*^ cells with a control lentiviral vector did not rescue cccDNA synthesis. Consistently, ELISA analysis for HBeAg in culture supernatant and immunofluorescence staining of intracellular HBcAg also confirmed that restoration of POLK expression in the HepG2-NTCP^*polk-/-*^ cells efficiently rescued not only cccDNA formation, but also viral gene transcription and protein expression (Fig 6C and 6D).

### POLL also plays a role in cccDNA formation

**Fig 7:**
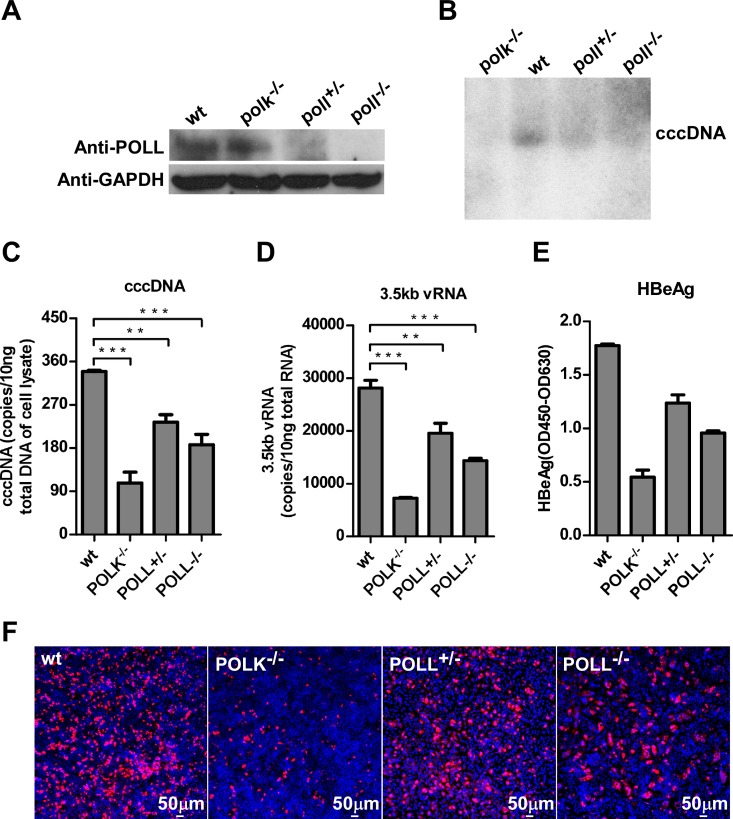
Depletion of POLL reduces HBV cccDNA synthesis. **(A)** POLL expression in parental HepG2-NTCP cells (wt), POLK deficient cells (HepG2-NTCP^*polk-/-*^) and HepG2-NTCP cell clones bearing disrupted *poll* gene in one (HepG2-NTCP^*poll+/-*^) or both alleles (HepG2-NTCP^*poll-/-*^) was determined by a Western blot assay. GAPDH protein served as a loading control. **(B-F)** The indicated HepG2-NTCP-derived cell lines were infected with HBV. HBV cccDNA on 7 dpi was detected by Southern blot (B). The amounts of cccDNA (C) and 3.5kb vRNA (D) on 7 dpi were measured by qPCR assays. Data were analyzed by an unpaired two-tailed *t* test. ** p<0.01 and *** p<0.001. Secreted HBeAg from the indicated HepG2-NTCP cells on 7 dpi was measured by ELISA (E). HBcAg in the infected cultures was stained with 1C10 mcAb (red). Nuclei were stained with DAPI (blue) (F). Images were captured with a Nikon A1-R confocal microscopy. Scale bars, 50μm.

Considering that knockout of POLK did not completely abolish HBV infection and cccDNA formation, and knockdown of POLL and POLH with siRNA also reduced HBV infection despite at a lesser extent, we further investigated the role of POLL in HBV infection with more rigorous experimental conditions. We accordingly generated POLL knockout cell lines with CRISPR/Cas9 technology. Infection of *poll* gene edited HepG2-NTCP^*poll+/-*^ and HepG2-NTCP^*poll-/-*^ cell clones (Fig 7A) with HBV demonstrated reduced levels of intracellular cccDNA (Fig 7B and 7C), 3.5kb vRNA (Fig 7D) and HBcAg (Fig 7F) as well as secreted HBeAg (Fig 7E) at day 7 post-infection, as compared to that in the parental HepG2-NTCP cells. However, the extent of POLL depletion on cccDNA formation and viral RNA and protein expression was less than that of POLK depletion. Taken together, our results strongly suggest that while POLK, POLL and POLH are each capable of supporting cccDNA synthesis at a different efficiency during a *de novo* HBV infection, POLK plays a more dominant role under the infection conditions examined in this study.

## Discussion

Synthesis of cccDNA is a critical, but not well-understood step in the life cycle of hepadnaviruses. Our current study characterized the kinetics of cccDNA formation in HBV infected cells and obtained strong evidence suggesting that cellular POLK plays a crucial role in cccDNA synthesis in *de novo* HBV infection. In addition, our findings reported herein also provide important clues for further investigation of viral and cellular factors in cccDNA biosynthesis and regulation.

### Establishment and regulation of cccDNA pool in virally infected hepatocytes

We demonstrated in this study that cccDNA is formed from incoming virion DNA in HepG2-NTCP cells at as early as 24 h post infection and establishes the pool size of approximately 3 copies of cccDNA per infected cell within a few days of infection. The kinetics of cccDNA accumulation as well as two lines of independent evidence obtained from HBV-ΔHBc infection of HepG2-NTCP cells and viral DNA polymerase inhibitor treatment of wild-type HBV-infected cells strongly support the notion that intracellular amplification does not play a significant role in the establishment of cccDNA pool in the HBV-infected hepatoma cells. This observation is consistent with the findings from HBV infection of primary human hepatocytes and HepaRG cells [38, 39], but distinct from DHBV infection of primary duck hepatocytes where significant intracellular cccDNA amplification occurs in a manner regulated by the level of its large envelope protein expression [19, 20]. However, intracellular amplification of cccDNA has been observed in HepG2-derived cell lines supporting constitutive or inducible HBV replication [40–42]. Developing therapeutics against chronic HBV infection requires better understanding the contribution of intracellular cccDNA amplification in the maintenance of persistent infection, and further investigation of the activity and regulation of this pathway in HBV-infected hepatocytes *in vivo* is thus warranted.

### Role of HBV core protein in cccDNA function

An elegant study by Chisari and colleagues demonstrated that although DHBV deficient for capsid protein expression (DHBVΔcp) infected primary duck hepatocytes and produced similar amounts of cccDNA from the incoming virions as did wild-type DHBV, the cccDNA in DHBVΔcp-infected hepatocytes was significantly less efficiently transcribed into viral RNAs, suggesting an important role of capsid protein in DHBV cccDNA transcription [43]. However, our results showed that HBV-ΔHBc infected HepG2-NTCP cells and expressed viral genes at a similar efficiency as wild-type HBV did. The results therefore suggest that the synthesis of HBV capsid protein may not significantly modify HBV cccDNA transcription activity. Interestingly, DHBV capsid protein is structurally distinct from the capsid proteins of mammalian hepadnaviruses [44] and may have HBx-like function in regulation of DHBV cccDNA transcription. However, the possibility that HBV capsid proteins from in-coming virions interact with cccDNA and promote its transcriptional activity cannot be completely ruled out. It has been shown that HBV capsid protein is a structural component of viral cccDNA minichromosome and its binding reduces the nucleosomal spacing of the minichromosome [45]. In addition, it has also been suggested that capsid protein promotes an epigenetic permissive state of HBV cccDNA by binding on CpG islands of cccDNA [46]. Of note, some non-nucleoside analogue compounds targeting capsid protein can dysregulate functional HBV capsid assembly [47–52]. Those capsid assembly effectors may alter the amounts and/or structure of cccDNA-bound capsid protein and consequentially interfere with cccDNA metabolism and function [53]. Intriguingly, interferon-stimulated gene (ISG) APOBEC3A seems to have a role in the destruction of cccDNA by direct interaction with HBV core protein [54]. Further investigation on the differential roles of capsid proteins in regulation of cccDNA function should shed light on this aspect of hepadnaviral pathobiology.

### Role of viral DNA polymerase in cccDNA formation

We showed herein that, similarly to DHBV and WHV, completion of plus strand DNA synthesis during *de novo* HBV infection of HepG2-NTCP cells is not sensitive to viral DNA polymerase inhibitors, suggesting the reaction is most likely catalyzed by a host DNA polymerase. In support of this hypothesis, by following the fate of viral DNA sequence during conversion of rcDNA into cccDNA, Seeger and colleagues demonstrated that independent of a viral enzymatic activity, a cellular DNA polymerase may fill in the 3’ end of both DNA strands [55]. The observed slight reduction of cccDNA amounts in ADV or ETV treated cells in this study could indicate either a minor contribution of viral DNA polymerase to cccDNA formation or an off-target inhibition of cellular functions required for cccDNA formation. Interestingly, studies of HBV cccDNA biosynthesis *via* intracellular amplification pathway in HepG2-derived stable cell lines, such as HepAD38 or HepDES19 cells, suggested that deproteinization and uncoating of progeny rcDNA require the completion of plus strand DNA synthesis, which requires viral DNA polymerase activity [41, 42]. Hence, different from rcDNA in virion particles with various length of incompletely synthesized plus-stranded DNA, the precursor rcDNA for cccDNA synthesis from the intracellular amplification pathway may have a very short gap in plus strand DNA and thus distinct DNA repair enzymes may be recruited to convert the rcDNA to cccDNA. Moreover, it had been shown that a small fraction of cccDNA can be formed from dslDNA *via* NHEJ DNA repair pathway. The cellular DNA polymerases required for cccDNA synthesis through intracellular amplification pathway and from dslDNA remain to be determined in future studies.

### Role of host cellular DNA polymerase in cccDNA biosynthesis and implications

While our results demonstrated that POLK plays a critical role in cccDNA formation during *de novo* HBV infection of cultured HepG2-NTCP, HepaRG and PTHs, we also showed that POLL and POLH play a role in cccDNA formation, albeit at a lesser extent. It is currently not clear whether each of the cellular DNA polymerases plays a redundant or distinct role in *de novo* cccDNA synthesis. As mentioned above, plus-strand DNA in rcDNA in virions has gaps of heterogeneous lengths. It is possible that depending on the length of gaps, distinct DNA repair complexes containing different repairing DNA polymerases are recruited to fill the gaps with different length. If this is the case, the three DNA polymerases may play non-redundant roles and be involved in conversion of distinct rcDNA precursors into cccDNA. Alternatively, each of the three cellular DNA polymerases may participate in a different DNA repair complex to fill the plus strand gaps, irrespective of their length, but in a different efficiency. These two possibilities will need to be further investigated.

POLK plays a functional role in nucleotide excision repair (NER) pathway by filling the gap produced upon excision of damaged nucleotides [56, 57]. The activity of POLK is partially dependent on the growth state of the cells, and reaches maximum activity under conditions of low deoxynucleotide concentration such as in non-dividing cells [56]. A previous study showed treatment of HBV-transfected HepG2 cells with aphidicolin arrested cells in the G1 phase could result in enhancement of cccDNA synthesis [58]. Consistent with this observation, we found that the efficiency of HBV infection closely correlates with the number of G0/G1 phase cells in HepG2-NTCP cultures. Therefore, HBV cccDNA formation may preferentially occur at G0/G1 phase of cell cycle, supporting the notion that HBV infects non-dividing cells, so that cccDNA is formed and stably exists in quiescent hepatocytes [59]. It is thus conceivable that cell cycle-dependent factor(s) or protein post translational modification affecting the physiologic state of hepatocytes may regulate the formation of HBV cccDNA.

Moreover, because cellular DNA polymerases must work in concert with other DNA repair proteins to restore the structure of damaged DNA, other DNA repair proteins in NER pathway may also play a role in HBV cccDNA formation. For example, it is possible that HBV hijacks cellular endonuclease (e.g. XPG) or exonuclease (e.g. Exo1) to cleave the capped RNA primer to leave a free 5’ end of plus strand DNA of rcDNA, and followed by POLK or other cellular DNA polymerase, such as POLL and POLH, to fill the gap using minus strand DNA as a template. Additionally, POLK has been shown to work together with POLD to fill in single stranded DNA gaps [56, 60] and XRCC1-Lig3 is required for ligation of NER-induced breaks in quiescent cells [61]; hence it will be interesting to test whether those host enzymes are involved in cccDNA formation.

In conclusion, taking advantage of highly efficient genetic manipulation of HepG2-NTCP HBV infection system, and in combination with studies using recombinant HBV virus and chemical inhibitors, we rigorously demonstrated that cellular DNA polymerase κ substantially contributes to HBV cccDNA formation in HepG2-NTCP cells. Our findings shed new light on the molecular mechanism of cccDNA formation and may facilitate the development of novel therapeutics to cure chronic hepatitis B.

## Materials and Methods

### Cells, antibodies and reagents

Human embryonic kidney cell 293T and human hepatoblastoma cell HepG2 were obtained from American Type Culture Collection (ATCC). Human hepatocellular carcinoma cell (Huh-7) was obtained from the Cell Bank of Type Culture Collection, Chinese Academy of Sciences. All the cell lines were maintained in Dulbecco’s Modification of Eagle’s Medium (DMEM; Life technologies) supplemented with 10% fetal bovine serum, 100 U/ml penicillin, and 100 μg/ml streptomycin at 37°C in a 5% CO_2_ incubator unless otherwise indicated. All experiments with HepG2 cells were carried out with cells grown on collagen-coated plates. HepaRG cells were obtained from Biopredic International (Rennes, France) and cultured according to the product manual. Differentiated HepaRG cells were obtained following a two-step procedure as previously described [38]. Primary Tupaia hepatocytes (PTHs) were obtained and cultured following a method as described previously [11].

Antibodies for human POLK (A-9) and C9 tag (1D4) were obtained from Santa Cruz Biotechnology. Antibody for human POLL (EPR7519(2)) was purchased from Abcam. Antibody against GAPDH, HRP-conjugated anti-mouse IgG and HRP-conjugated anti-rabbit IgG were purchased from Sigma. Alexa Fluor 488- and 546-conjugated anti-mouse IgG antibodies were purchased from Life Technologies. Other mouse antibodies to detect the Core protein (1C10) and surface antigen (17B9) of HBV, HDV delta antigen (4G5) were described previously [11, 62]. N-terminally myristoylated peptide of the N-terminal 47 amino acid residues of pre-S1 domain of HBV strain S472 (Accession number: EU554535.1), myr-47, was synthesized by SunLight peptides Inc.. Adefovir (ADV), entecavir (ETV), aphidicolin (APH) and other general chemicals were purchased Sigma unless otherwise stated.

### Plasmids, siRNA oligos and transfection

To construct human POLK expression plasmids, a full-length human POLK cDNA was cloned from HepG2 mRNA into a modified pLKO.1-puro lentiviral vector under the control of a CMV promoter for stable expression in POLK knockout HepG2-NTCP cells. An N-terminal GFP-tagged POLK expression plasmid (GFP-POLK-wt) was constructed for investigating POLK subcellular localization and RNAi rescue experiment. For the single siRNA-mediated gene knockdown experiments, siRNA transfection was performed using Lipofectamine RNAiMAX (Life technologies) according to the manufacturer’s instruction. HepG2-NTCP cells in 48-well plates were transfected with 5 pmol siRNA and 0.5 μl Lipofectamine RNAiMAX in 25 μl Opti-MEM and were challenged with the HBV (or HDV, VSV as indicated) at 72 h post transfection. For combined dual RNAi experiments, HepG2-NTCP cells in 48-well plates were transfected with individual siRNAs for indicated two target genes (each 2.5 pmol, total 5pmol) and 0.5 μl Lipofectamine RNAiMAX in 25 μl Opti-MEM and were challenged with the HBV (or HDV, VSV as indicated) at 72 h post transfection. The siRNA target sequences used in this study are shown in S1 Table, which have been confirmed to be functional in other previous studies [36]. siRNAs targeting *Tupaia polk* gene were designed by RNAi designer (https://rnaidesigner.thermofisher.com/rnaiexpress/). The specificities of siRNAs were confirmed using a BLAST search. The efficiency of gene knockdown was determined by qPCR or Western blot assays. The qPCR primers used for quantification of human POLK, POLH and GAPDH mRNA expression are listed below. Human POLK: qPOLK-F: 5'-CCAGACATCACAACCATTCC and qPOLK-R: 5'-TCAAGGCTTCCAGACTGATG; human POLH: qPOLH-F: 5'-GTGCCAGTTACCAGCTCAGA and qPOLH-R: 5'-AGGTAATGAGGGCTTGGATG; GAPDH: 5’-GAAGGTGAAGGTCGGAGTCA (forward) and 5’-TGGAATCATATTGGAACATGT (reverse). Both human POLK and POLH mRNA levels are normalized to the expression level of GAPDH, respectively. For Tupaia POLK mRNA measurements, qtsPOLK-F: 5'-TCACTAGCCAGCAGCTAAGGAAAGC and qtsPOLK-R: 5'-CATGCTCATTGATCCTACAGCAATG were used as qPCR primers. 5’-GTGAAGGTCGGAGTAAACG (forward) and 5’-CCATGGGTGGAGTCATACT (reverse) were used for Tupaia GAPDH, the mRNA expression level of tsPOLK is normalized by tsGAPDH. All siRNA oligos were synthesized by NIBS biological resources facility. All transfections were conducted in duplicates. Cytotoxic effects of siRNA were examined using alamarBlue reagents (Life technologies).

### Virus production, *in vitro* viral infection and inhibition assays

HBV genotype D virus and HDV genotype I virus were produced by transient transfection of Huh-7 cells with the corresponding plasmids as described previously [11]. HBc protein deficient virus (HBV-ΔHBc) was generated by co-transfection of Huh-7 cells with a plasmid harboring 1.05 copies of HBV genome with a stop codon at the 38^th^ codon (Y) of core gene open reading frame, and an intact HBc protein expression vector. Virus stocks were aliquoted and stored at -80°C. HBV and HDV infection assays have been described previously [11]. Briefly, HepG2-NTCP cells were firstly cultured in 48-well plates with DMEM complete medium for 3–4 h, then the cells were cultured with PMM medium for another 20 hrs. The cells were then infected with a multiplicity of 100 genome equivalents (mge) of HBV or 500 mge of HDV in the presence of 4% PEG8000 for 24 hrs at 37°C. Cells were maintained in PMM with regular medium changing every other day. For viral inhibition assay, 100 nM myr-47 lipopeptide was pre-incubated with HBV viruses before adding to HepG2-NTCP cells; chemicals were pre-incubated with HepG2-NTCP cells at 37°C for 12 h before virus inoculation.

To generate VSV-G protein pseudotyped lentiviral particles expressing EGFP, PLKO.3G plasmid together with pSPAX2 and pMD2G were co-transfected into 293T cells at the ratio of 4:3:1 (PLKO.3G:pSPAX2:pMD2G) using Lipofectamine 2000 (Life technologies). Forty-eight hours after transfection, supernatants were harvested, cleared by centrifugation and stored at -80°C. Human POLK or gRNA expressing lentivirus was generated using a similar protocol as that described above but replacing the pLKO.3G plasmid with the pLKO.1-CMV-POLK-puro or pLKO.1-gRNA-EGFP plasmid, respectively. Infection of cells with lentiviral pseudovirus was performed as described previously [63]. Virus-related experiments were conducted in a BSL-2 facility at the National Institute of Biological Sciences, Beijing.

### qRT-PCR analysis of viral nucleic acids

HBV viral particles were purified using Nycodenz gradient ultracentrifugation of the culture supernatants of Huh-7 cells transfected with plasmid for HBV production. The HBV DNA levels of the virus fractions were quantified using specific primers: 5’-GAGTGTGGATTCGCACTCC (forward) and 5’-GAGGCGAGGGAGTTCTTCT (reverse) by real-time PCR using SYBR Premix Ex Taq kit (TaKaRa) on an ABI 7500 Fast Real-Time system instrument (Applied Biosystems, United States). The viral genome equivalent copies were calculated based on a standard curve generated with known copy numbers.

For analysis of HBV 3.5kb viral RNA, total RNA from infected cells was extracted by TRIzol reagent (Life technologies), 0.5 μg of total RNA was digested with DNase I (Life technologies) and reverse-transcribed into cDNA using PrimerScript RT Reagent Kit (TaKaRa) in a 20 μl reaction. Real time-PCR analysis was performed using the SYBR Premix Ex Taq on ABI 7500 Fast Real-Time PCR System. HBV 3.5kb viral RNA copy numbers were deduced from a standard curve generated from known nucleic acid quantities. The mRNA level of HBV 3.5kb viral RNA was normalized to that of GAPDH mRNA. The primers used for each gene examined are listed below. HBV 3.5kb viral RNA: 5'-GAGTGTGGATTCGCACTCC (forward) and 5'-GAGGCGAGGGAGTTCTTCT (reverse); GAPDH mRNA: 5’-GAAGGTGAAGGTCGGAGTCA (forward) and 5’-TGGAATCATATTGGAACATGT (reverse).

For quantification of HBV cccDNA, infected cells were lysed in a lysis buffer (20 mM Tris, 0.4 M NaCl, 5 mM EDTA, 1% SDS, pH = 8.0) in the presence of proteinase K (QIAGEN), total DNA was extracted according to a standard phenol-chloroform extraction protocol. 500 ng of total DNA was digested with 0.5 μl plasmid-safe adenosine triphosphate (ATP)-dependent deoxyribonuclease DNase (PSAD) (Epicentre Technologies) in 25 μl reaction for 8 h at 37°C to allow removal of linear genomic DNA and HBV replication intermediates (rcDNAs, single-stand DNAs, linear double-strand DNAs). DNase was inactivated by incubating the reactions for 30 min at 70°C. 20 ng of digested DNA was used for quantification of HBV cccDNA, 5'-TGCACTTCGCTTCACCT (forward) and 5'-AGGGGCATTTGGTGGTC (reverse) were used as HBV cccDNA specific primers, the real-time PCR was performed using the SYBR Premix Ex Taq on ABI 7500 Fast Real-Time PCR System as the following reaction procedure: 95°C for 5 min then 45 cycles of 95°C for 30 s, 62°C for 25 s, and 72°C for 30 s. The amount of HBV cccDNA in a DNA preparation was determined by real-time PCR using a plasmid containing HBV-D genome as the standard. The pool size of HBV cccDNA per infected cell was calculated by quantification of cccDNA copies using digital PCR in the whole cell population and estimation of the number of infected cells by immunostaining of intracellular HBcAg.

### Southern blot analysis of HBV cccDNA and intracellular viral core DNA

Selective extraction of HBV cccDNA from HBV infected cells was achieved by a modified Hirt method as previously described [64, 65]. Briefly, infected cells from one well of 6-well plates were lysed in Hirt lysis buffer (10 mM Tris-HCl, 10 mM EDTA, 0.6% SDS, pH = 7.4) for 30 min at room temperature. After adding 5 M NaCl, the cell lysate was vigorously mixed and incubated at 4°C overnight. After centrifugation at 10,000 rpm for 30 min at 4°C, the supernatant was extracted twice with saturated Tris-phenol (pH = 8.0) and once with phenol:chloroform. The extracted DNA was precipitated with equal volumes of isopropanol at -20°C overnight. The DNA pellet was washed with 70% ethanol and dissolved in TE buffer (10 mM Tris-HCl, 1mM EDTA, pH = 8.0), and digested with *Hind*III or *EcoR*I restriction enzyme (NEB) before being analyzed.

For detection of cccDNA by Southern blot, the extracted HBV cccDNA sample was subjected to 1.2% agarose gel electrophoresis and transferred onto Amersham Hybond-N^+^ membrane (GE Healthcare). The Hybond-N^+^ membrane was crosslinked in a UV crosslinker chamber with UV energy dosage at 1200 mJ and followed by being probed with [α-^32^P]dCTP (250 μCi, Perkin Elmer)-labeled HBV genotype D (Accession number: U95551.1) linear full-length genomic DNA. Hybridization was performed in Perfecthyb plus hybridization buffer (Sigma) with prehybridization for 1 h at 65°C and overnight hybridization at 65°C, followed by two washes in wash buffer (0.1×SSC, 0.1% SDS) at 65°C. The membrane was exposed to Carestream X-OMAT BT Film (XBT-1, Carestream). 100 pg each of 3.2kb, 2.1kb and 1.7kb HBV DNA fragments prepared by PCR amplification of a plasmid containing 1.0 copies linear HBV genotype D genome was used as DNA marker.

### ELISA assay for HBV viral antigens

HBeAg and HBsAg from supernatants of HBV infected cells were measured using ELISA kits (Wantai Pharm Inc. Beijing, China) by following the manufacturer’s instructions. Supernatants from HBV infected cells were harvested at each time point examined in the various assays and were diluted 2-fold with PMM before ELISA. All experiments were performed in duplicates and repeated at least two times independently.

### Immunofluorescence assay

Virus infected cells in 48-well plates were washed three times with pre-cooled PBS and fixed by 4% paraformaldehyde for 10 min, followed by permeablization for 10 min at room temperature with 0.5% Triton X-100. After incubation for 1 h with 3% BSA for blockade of nonspecific binding, primary antibodies were added for incubation for 1 h at 37°C. The bound antibodies were visualized by incubation with secondary antibodies (Alexa Fluor 488 donkey anti-mouse IgG or Alexa Fluor 546 anti-mouse IgG). Images were acquired using a Nikon A1-R confocal microscope or a Nikon Eclipse Ti Fluorescence Microscopy.

### Generation of transgene stable expression and knockout cell lines

The POLK rescue cell lines were generated by infection of POLK stable knockout cells with recombinant lentivirus expressing POLK, and cells were selected with puromycin (Sigma). Expression of POLK in the established stable cell lines were verified by Western blot assay.

To generate POLK deficient HepG2-NTCP cells, genomic engineering of *polk* gene was achieved with the CRISPR/Cas9 system as described with the following single-guide (sg) RNA target sequences: CTTCTCCTTTGTGCTATCCA (sgPOLK-1), GATGATCTTCTGCTTAGGAT (sgPOLK-2). Firstly, stably expressing Cas9 cell line (HepG2-NTCP/Cas9) was generated by transfection of human codon-optimized Cas9 (hCas9) expressing vector (Addgene) using Lipofectamine 2000 and selection with Blasticidin (Calbiochem). Then the cells were infected with gRNA expressing lentivirus. After culture for 3 d *in vitro*, EGFP positive cells were sorted by flow cytometry FACSAria II, and further analyzed by T7 endonuclease I (NEB) assay. Genomic DNA sequence of POLK around the gRNA targeting site was amplified using the following primers: cas9-POLK-F: 5'-GTGTCGAACCCCTGAGCTCAGTCAATCT and cas9-POLK-R: 5'-AGGTGAACAGGAACATATACATTATTT. Single clones of sorted cells were obtained by serial dilutions and amplified, verified by sequencing of PCR fragments, and confirmed by Western blot using anti-POLK antibody. POLL deficient HepG2-NTCP cell lines were constructed by following the above mentioned procedure, a 20-bp single-guide sequence (sgPOLL: CGGGCCCATGTTGTGCGCAC) targeting DNA within the third exon of *poll* gene was selected. cas9-POLL-F: 5’-GCTATATGTAGAAGGAAAGCTGTC and cas9-POLL-R: 5’-ACTGGGATCAGCCCACCTACTGG were used as primers for amplification of a region around the gRNA targeting site and the PCR products were further analyzed by T7E1 assay. Individual clones were validated by sequencing of PCR fragments, and confirmed by Western blot using anti-POLL antibody.

## Supporting Information

S1 Fig Southern blot analysis of cccDNA from HBV infected HepG2-NTCP cells. HBV cccDNA was extracted from one well of 6-well plates of HBV infected HepG2-NTCP cells at dpi 7 by Hirt method and followed by digestion with *Hind*III or *EcoR*I restriction enzyme, the DNA samples were analyzed by Southern blot with an [α-^32^P]dCTP-labeled full-length HBV DNA probe. HBV virion DNA extracted from pelleted viral particles by 8% PEG8000 served as a control; 100 pg each of 3.2kb, 2.1kb and 1.7kb HBV DNA fragments were used as molecular weight markers. DslDNA: double strand linear DNA; cccDNA: covalently closed circular DNA; rcDNA: relaxed circular DNA.
(TIF)

S2 Fig Production of HBc deficient HBV virus. **(A and B)** Schematic representation of HBV-ΔHBc virus production in Huh-7 cells and subsequent quantification of HBV DNA (A). The pure culture supernatant of Huh-7 cells transfected with plasmids for HBV-ΔHBc production was applied to preformed discontinuous density gradients of Nycodenz (1.15, 1.18, 1.21, 1.24 and 1.27 g/ml, among which the 1.21 fraction was prepared with 240μl virus-containing culture medium and 560μl Nycodenz stock), the volume of each density gradient of Nycodenz was 800μl. After centrifugation (4°C; 30,000 rpm; 16 h; Beckman MLS-50 rotor), 20 fractions (200μl each) were collected from the top of the tube, 2μl of each fraction was subjected to quantify the levels of HBV DNA by qPCR assay (B). The fractions of HBV virion and capsid were distinguished based on their different physical properties, the density of virion is around 1.18 in Nycodenz solution.
(TIF)

S3 Fig Effects of adefovir and entecavir on HBV cccDNA formation in HBV infected HepG2-NTCP cells. HepG2-NTCP cells were treated with indicated drugs and harvested as depicted in Fig 2D. The effects of ADV and ETV on HBV cccDNA biosynthesis in HepG2-NTCP cells infected by wild-type HBV was determined by Southern blot (left panel). The intensity of HBV cccDNA bands were determined by Image J and the relative amounts of cccDNA were expressed as the percentage of that in the mock-treated control. The intracellular cccDNA were also quantified by a qPCR (right panel). Data is representative of three independent experiments. Data were analyzed by an unpaired two-tailed *t* test. NS: non-significant, * p<0.05 and ** p<0.01.
(TIF)

S4 Fig Measurement of cell viability after siRNA transfection. Cytotoxicity of each siRNA on HepG2-NTCP cells was determined by measuring metabolic activity of the cells using alamarBlue cell viability reagent by following the manufacturer’s instruction. Data are presented as Mean ± SD (n = 2).
(TIF)

S5 Fig siRNA-mediated single and dual knockdown of POLK, POLL and POLH genes inhibited HBV infection. **(A-D)** HepG2-NTCP cells were transfected with siRNA targeting POLL, or a negative control siRNA (NC). On three days post siRNA transfection, cells were lysed with RIPA buffer supplemented with a protease inhibitor cocktail. The same amounts of total cell lysates were subjected to Western blot analysis with antibodies against POLL or GAPDH (A, left). HepG2-NTCP cells were transfected with siRNA targeting POLH, or a negative control siRNA (NC). Three days later, total RNA was extracted and reverse-transcribed into cDNA, the mRNA level of POLH was determined by qPCR and normalized to GAPDH expression, respectively (A, right). The relative expression of POLH in NC treated sample was set at 1.0. Mean and SD are presented from three independent measurements. Data were analyzed by an unpaired two-tailed *t* test, *** p<0.001. The indicated siRNA (5pmol) transfected HepG2-NTCP cells were infected with HBV at 3 days post siRNA transfection. On 7 dpi, HBV 3.5kb vRNA levels were quantified by qPCR assays (B). Secreted HBeAg was measured by ELISA (C). Intracellular HBcAg expression was detected by immunostaining (D). HBcAg was stained with 1C10 mcAb (green). Nuclei were stained with DAPI (blue). Images were examined using a Nikon A1-R confocal microscopy. Scale bars, 50μm. **(E-G)** HepG2-NTCP cells were transfected with dual siRNAs targeting the indicated genes with a total amount of 5 pmol (2.5 pmol each), and the cells were infected with HBV at 3 days post siRNA transfection. HBV 3.5kb vRNA levels on 7 dpi were quantified by qPCR assays (E). Secreted HBeAg at 7 dpi were measured by ELISA (F). Intracellular HBcAg expression on 7 dpi was detected by immunostaining (G). HBcAg was stained with 1C10 mcAb (green). Nuclei were stained with DAPI (blue). Images were examined using a Nikon A1-R confocal microscopy. Scale bars, 50μm.
(TIF)

S6 Fig Effects of aphidicolin on cccDNA formation in HepG2-NTCP cells infected by HBV. HepG2-NTCP cells were pre-treated with indicated doses of aphidicolin (APH) for 12 hrs and followed by being inoculated with HBV in the presence of APH for 24 hrs. The infected cells were cultured in PMM with APH for additional 7 days. HBV cccDNA was extracted by Hirt method and analyzed by Southern blot. The intensity of HBV cccDNA bands were determined by Image J and the relative amounts of cccDNA were expressed as the percentage of that in the mock-treated control (A), the levels of secreted HBeAg and HBsAg were assessed by ELISA (B).
(TIF)

S7 Fig Knocking down POLK does not affect HDV and lentivirus-VSV-GFP infection. HepG2-NTCP cells were transfected with siRNA targeting NTCP or POLK or with a scramble siRNA as negative control (NC). Three days post siRNA transfection, the cells were inoculated with HDV for 24 h or lentivirus-VSV-GFP for 8 h. HDV infection was detected by immunostaining for HDV delta antigen with 4G5 antibody (red). Images were examined using a Nikon A1-R confocal microscopy; scale bars, 50μm (A). Lentivirus-VSV-GFP infection was recorded by fluorescence microscope on 3 dpi (B).
(TIF)

S8 Fig Ectopic expression of POLK rescues HBV infection in POLK knockdown HepG2-NTCP cells. **(A)** HepG2-NTCP cells were infected with lentivirus encoding wild-type POLK (GFP-POLK-wt), or a siPOLK-1 resistant POLK (GFP-POLK-res), or GFP alone (Vector), respectively. Eight hours post lentiviral transduction, the cells were transfected with siPOLK-1. Ectopic expression of POLK was determined by fluorescence microscope 72 hrs after the transfection of siPOLK-1 (upper panel); The expression and localization of GFP-POLK-wt was analyzed by confocal microscopy (lower panel); nuclei were stained with DAPI (blue). **(B-D)** The HepG2-NTCP cells were infected with HBV 72 hrs after siRNA transfection. On 7 dpi, intracellular cccDNA (B) and 3.5kb vRNA (C) levels were quantified by qPCR assays. Secreted HBeAg was measured by ELISA (D). The data is representative of three independent experiments. Data were analyzed by an unpaired two-tailed *t* test. * p<0.05, ** p<0.01 and *** p<0.001.
(TIF)

S9 Fig Generation of POLK and POLL gene knockout HepG2-NTCP cell lines. **(A)** Schematic representation of the single-guide (sg) RNA targeting sites at exon 2 of human *polk* gene (top) and at exon 3 of human *poll* gene (bottom). The sgRNA coding sequence is labeled in orange. The protospacer-adjacent motif (PAM) sequence is labeled in blue. **(B)** The procedure for generation of POLK, or POLL knockout HepG2-NTCP cells using CRISPR/Cas9 system is depicted. **(C)** DNA fragments containing sgRNA targeting region were amplified by PCR from genomic DNA of sgRNA transduced cells, the indel mutations were detected by T7E1 digestion of targeted PCR fragment. The left is for *polk* gene, the right is for *poll* gene. **(D)** Sequence alignment of wild-type *polk* gene (top), or *poll* gene (bottom) and the mutated alleles induced by sgRNA/Cas9 in isolated individual clonal cell lines, respectively. The wild-type sequence is shown at the top and deletions are shown as dashed lines.
(TIF)

S10 Fig Analyses of the integrity and function of NTCP in the POLK knockout stable cell lines. **(A)** Immunoblot analysis of NTCP expression in the indicated cell lines. GAPDH served as loading control. **(B)** [^3^H] taurocholate uptake efficiency was quantified by scintillation counting. HepG2 cell line was used as a negative control. Data are presented as Mean ± SD and representative of at least three independent replications. Data were analyzed by an unpaired two-tailed *t* test. NS: non-significant and *** p<0.001. **(C)** Parental HepG2-NTCP and the indicated HepG2-NTCP-derived cell lines were infected with HDV, the efficiency of HDV infection was determined by immunostaining for HDV delta antigen with 4G5 antibody (red) on 7 days post infection. Nuclei were stained with DAPI (blue). Images were examined using a Nikon A1-R confocal microscopy. Scale bars, 50μm.
(TIF)

S1 Table Targeting sequences of siRNAs used in the study. (PDF)
