# Supplementary figures and images for: DNA Polymerase κ Is a Key Cellular Factor for the Formation of Covalently Closed Circular DNA of Hepatitis B Virus
Source: PLoS Pathog. 2016 Oct 26;12(10):e1005893. doi: 10.1371/journal.ppat.1005893 (PMC5081172; doi:10.1371/journal.ppat.1005893)

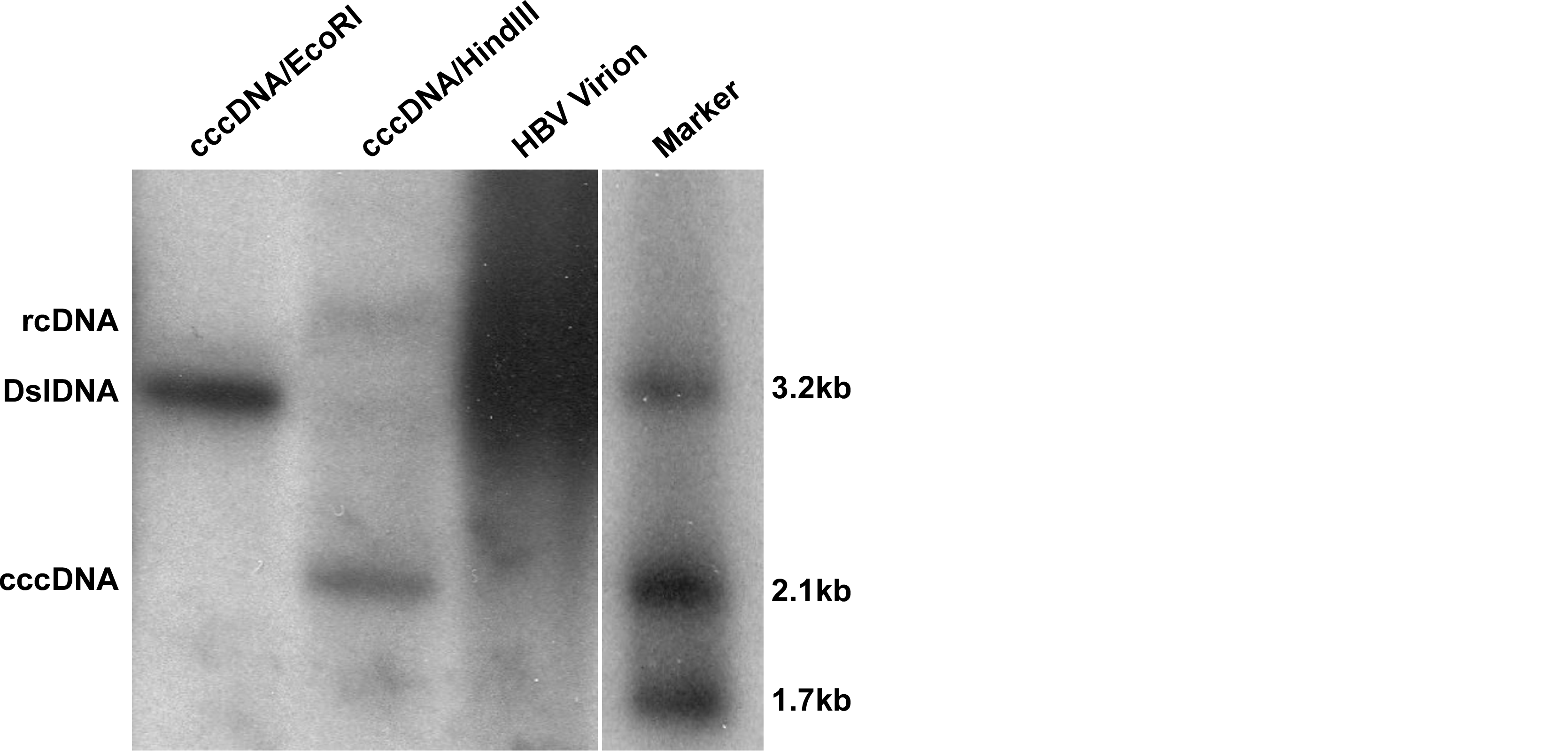

Supplement: S1 Fig — HBV cccDNA was extracted from one well of 6-well plates of HBV infected HepG2-NTCP cells at dpi 7 by Hirt method and followed by digestion with HindIII or EcoRI restriction enzyme, the DNA samples were analyzed by Southern blot with an [α-32P]dCTP-labeled full-length HBV DNA probe. HBV virion DNA extracted from pelleted viral particles by 8% PEG8000 served as a control; 100 pg each of 3.2kb, 2.1kb and 1.7kb HBV DNA fragments were used as molecular weight markers. DslDNA: double strand linear DNA; cccDNA: covalently closed circular DNA; rcDNA: relaxed circular DNA. (TIF) [file ppat.1005893.s001.tif]

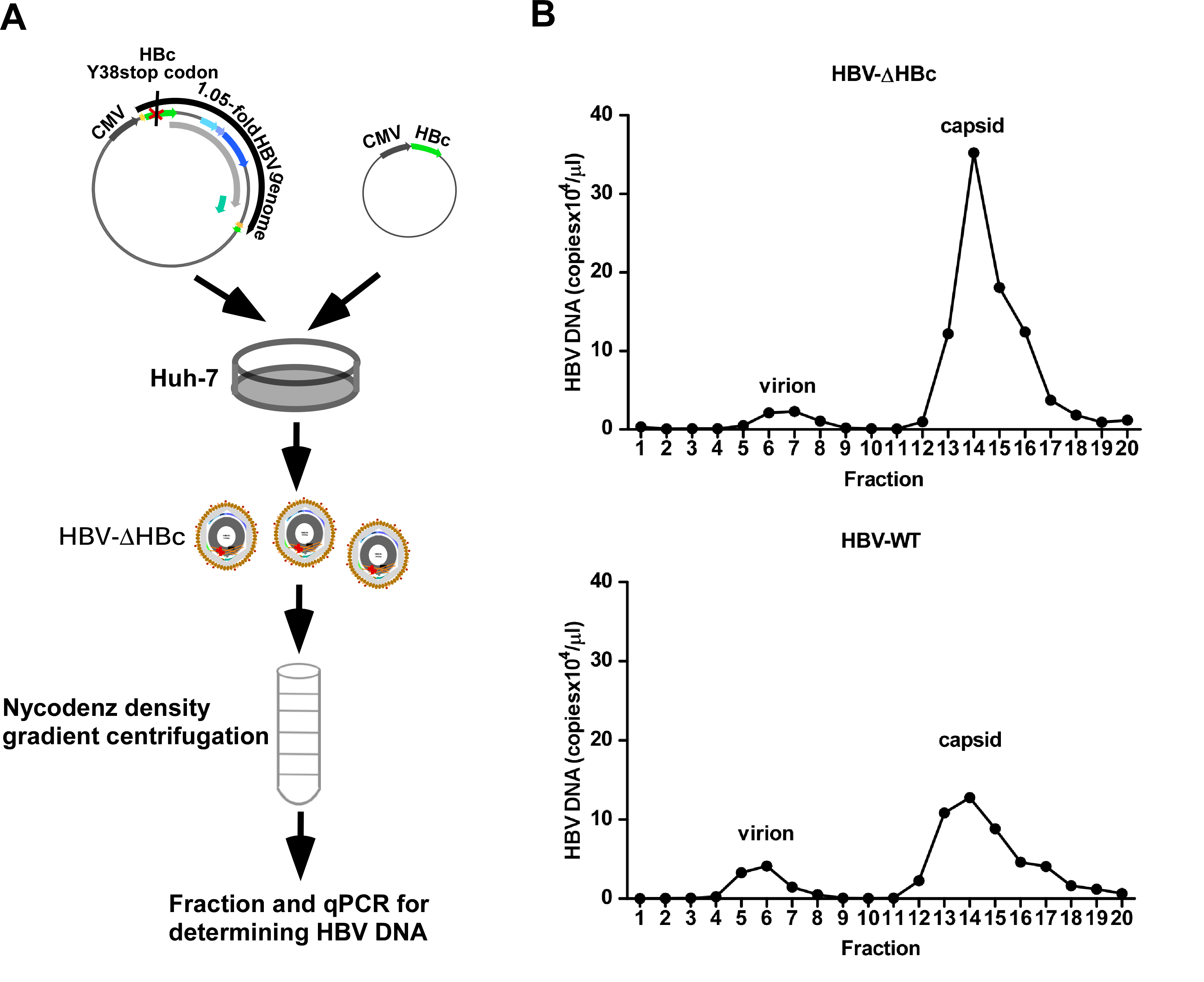

Supplement: S2 Fig — (A and B) Schematic representation of HBV-ΔHBc virus production in Huh-7 cells and subsequent quantification of HBV DNA (A). The pure culture supernatant of Huh-7 cells transfected with plasmids for HBV-ΔHBc production was applied to preformed discontinuous density gradients of Nycodenz (1.15, 1.18, 1.21, 1.24 and 1.27 g/ml, among which the 1.21 fraction was prepared with 240μl virus-containing culture medium and 560μl Nycodenz stock), the volume of each density gradient of Nycodenz was 800μl. After centrifugation (4°C; 30,000 rpm; 16 h; Beckman MLS-50 rotor), 20 fractions (200μl each) were collected from the top of the tube, 2μl of each fraction was subjected to quantify the levels of HBV DNA by qPCR assay (B). The fractions of HBV virion and capsid were distinguished based on their different physical properties, the density of virion is around 1.18 in Nycodenz solution. (TIF) [file ppat.1005893.s002.tif]

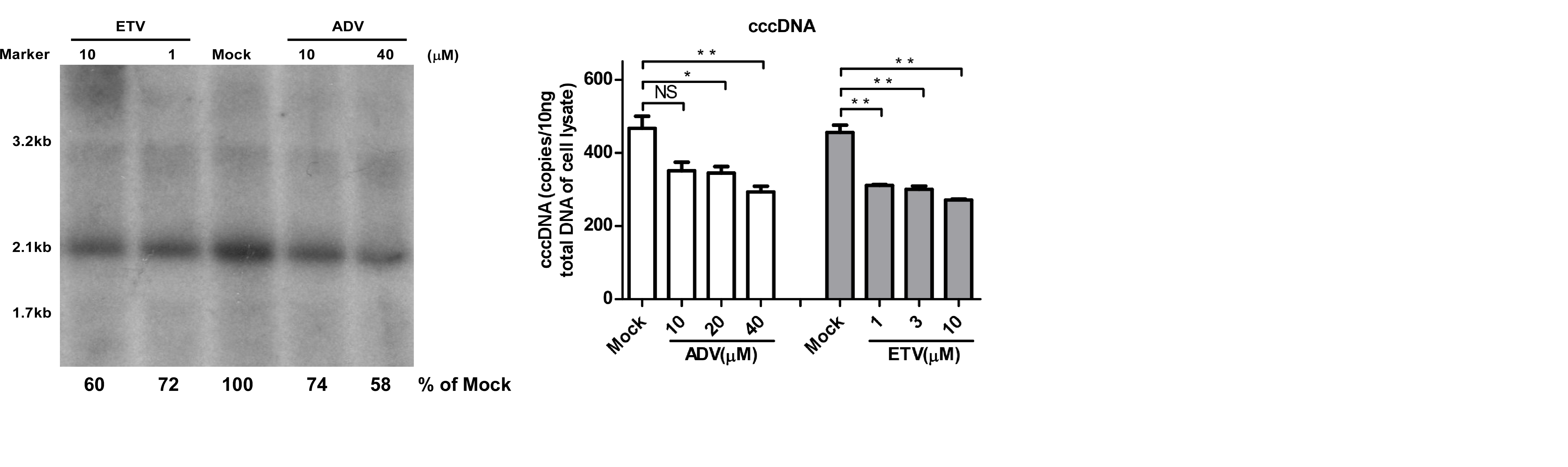

Supplement: S3 Fig — HepG2-NTCP cells were treated with indicated drugs and harvested as depicted in Fig 2D. The effects of ADV and ETV on HBV cccDNA biosynthesis in HepG2-NTCP cells infected by wild-type HBV was determined by Southern blot (left panel). The intensity of HBV cccDNA bands were determined by Image J and the relative amounts of cccDNA were expressed as the percentage of that in the mock-treated control. The intracellular cccDNA were also quantified by a qPCR (right panel). Data is representative of three independent experiments. Data were analyzed by an unpaired two-tailed t test. NS: non-significant, * p<0.05 and ** p<0.01. (TIF) [file ppat.1005893.s003.tif]

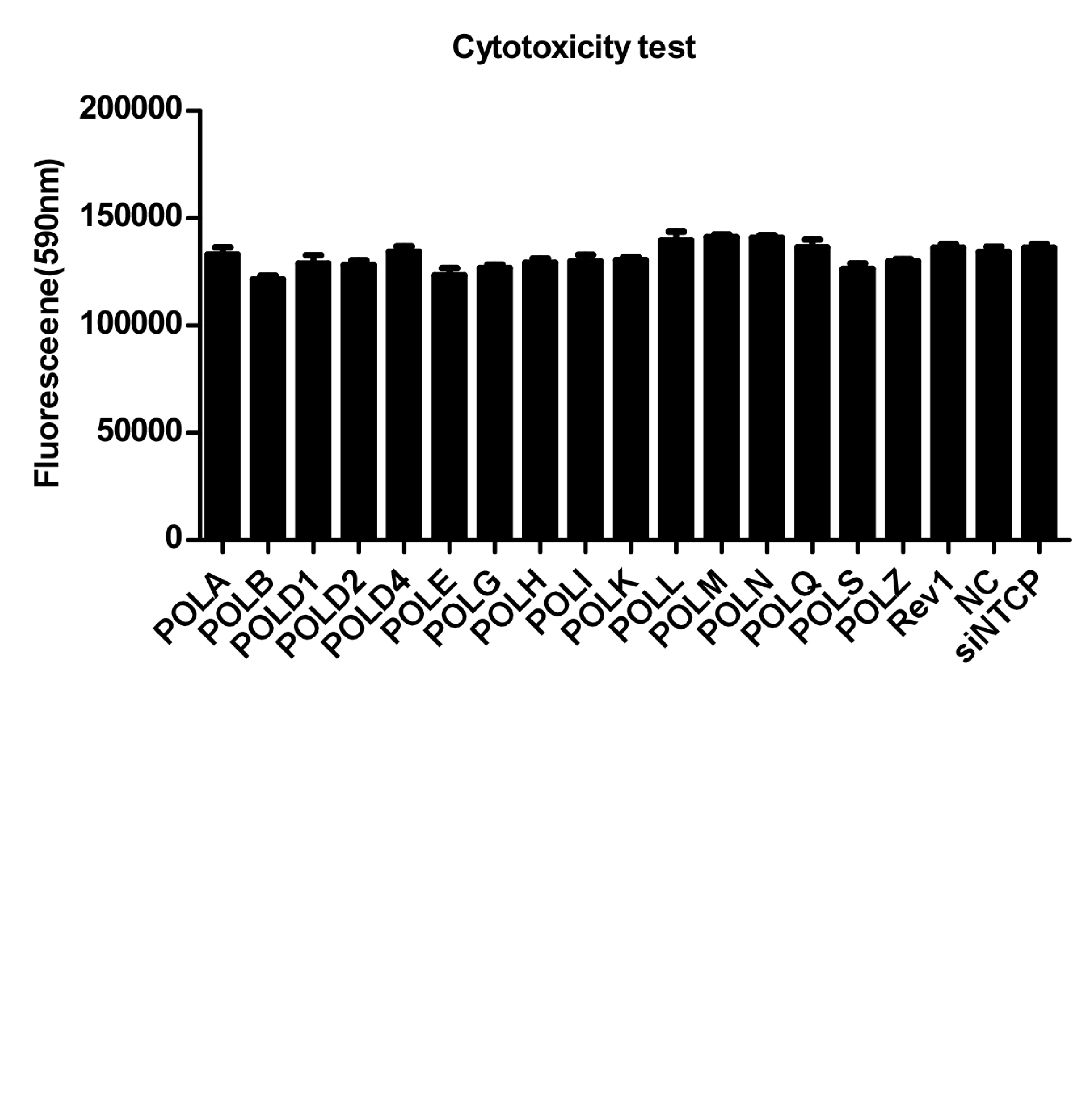

Supplement: S4 Fig — Cytotoxicity of each siRNA on HepG2-NTCP cells was determined by measuring metabolic activity of the cells using alamarBlue cell viability reagent by following the manufacturer’s instruction. Data are presented as Mean ± SD (n = 2). (TIF) [file ppat.1005893.s004.tif]

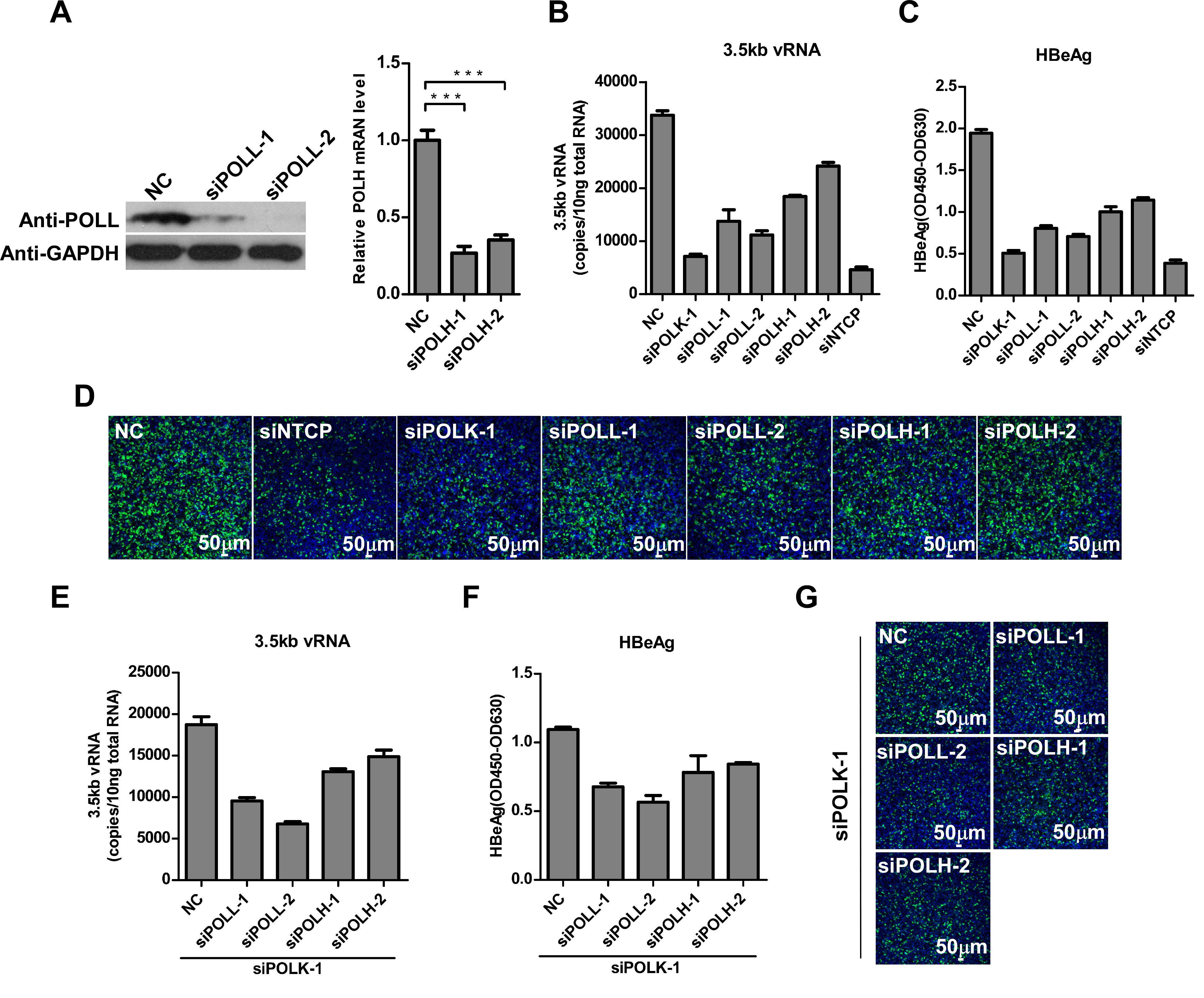

Supplement: S5 Fig — (A-D) HepG2-NTCP cells were transfected with siRNA targeting POLL, or a negative control siRNA (NC). On three days post siRNA transfection, cells were lysed with RIPA buffer supplemented with a protease inhibitor cocktail. The same amounts of total cell lysates were subjected to Western blot analysis with antibodies against POLL or GAPDH (A, left). HepG2-NTCP cells were transfected with siRNA targeting POLH, or a negative control siRNA (NC). Three days later, total RNA was extracted and reverse-transcribed into cDNA, the mRNA level of POLH was determined by qPCR and normalized to GAPDH expression, respectively (A, right). The relative expression of POLH in NC treated sample was set at 1.0. Mean and SD are presented from three independent measurements. Data were analyzed by an unpaired two-tailed t test, *** p<0.001. The indicated siRNA (5pmol) transfected HepG2-NTCP cells were infected with HBV at 3 days post siRNA transfection. On 7 dpi, HBV 3.5kb vRNA levels were quantified by qPCR assays (B). Secreted HBeAg was measured by ELISA (C). Intracellular HBcAg expression was detected by immunostaining (D). HBcAg was stained with 1C10 mcAb (green). Nuclei were stained with DAPI (blue). Images were examined using a Nikon A1-R confocal microscopy. Scale bars, 50μm. (E-G) HepG2-NTCP cells were transfected with dual siRNAs targeting the indicated genes with a total amount of 5 pmol (2.5 pmol each), and the cells were infected with HBV at 3 days post siRNA transfection. HBV 3.5kb vRNA levels on 7 dpi were quantified by qPCR assays (E). Secreted HBeAg at 7 dpi were measured by ELISA (F). Intracellular HBcAg expression on 7 dpi was detected by immunostaining (G). HBcAg was stained with 1C10 mcAb (green). Nuclei were stained with DAPI (blue). Images were examined using a Nikon A1-R confocal microscopy. Scale bars, 50μm. (TIF) [file ppat.1005893.s005.tif]

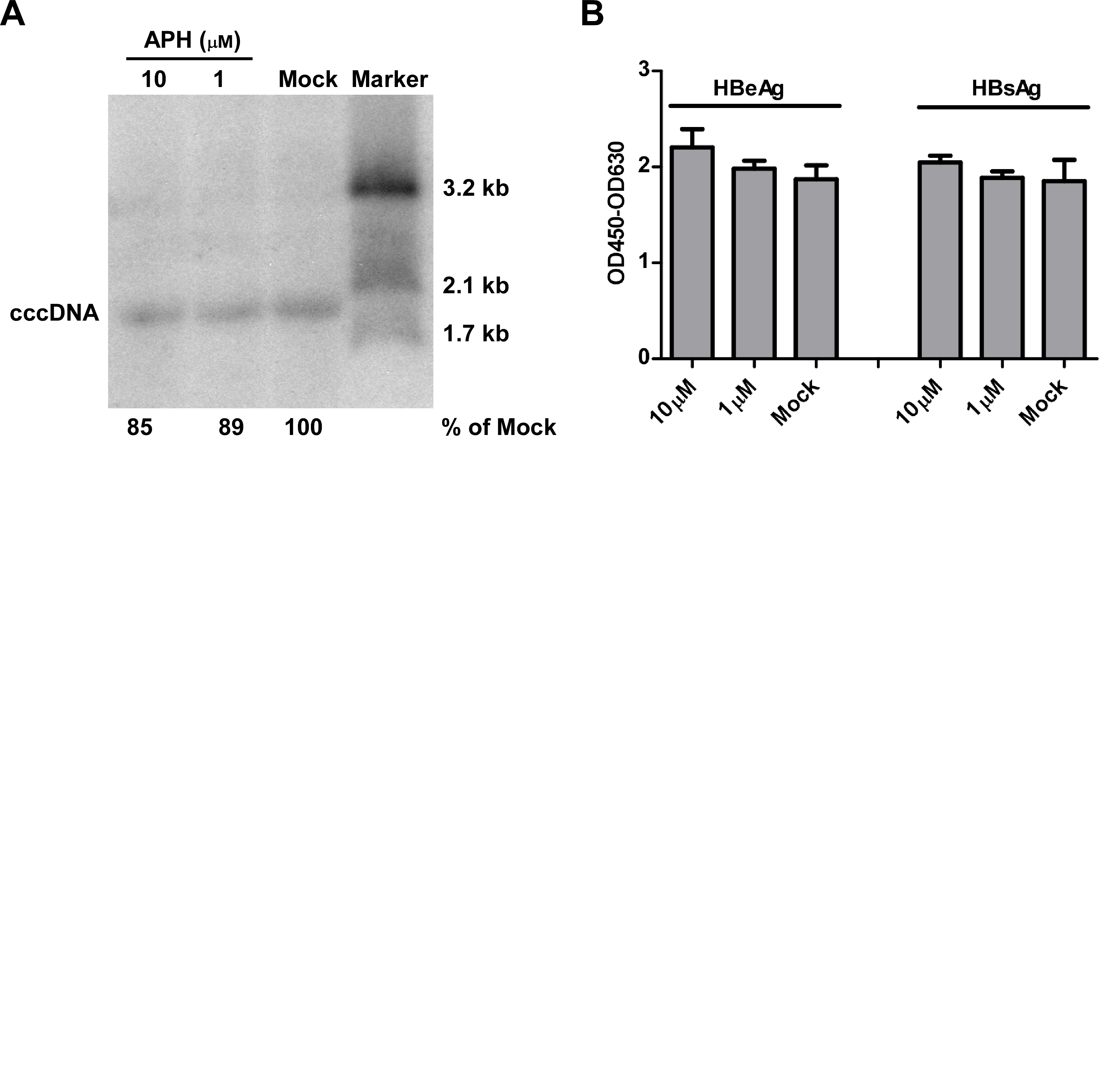

Supplement: S6 Fig — HepG2-NTCP cells were pre-treated with indicated doses of aphidicolin (APH) for 12 hrs and followed by being inoculated with HBV in the presence of APH for 24 hrs. The infected cells were cultured in PMM with APH for additional 7 days. HBV cccDNA was extracted by Hirt method and analyzed by Southern blot. The intensity of HBV cccDNA bands were determined by Image J and the relative amounts of cccDNA were expressed as the percentage of that in the mock-treated control (A), the levels of secreted HBeAg and HBsAg were assessed by ELISA (B). (TIF) [file ppat.1005893.s006.tif]

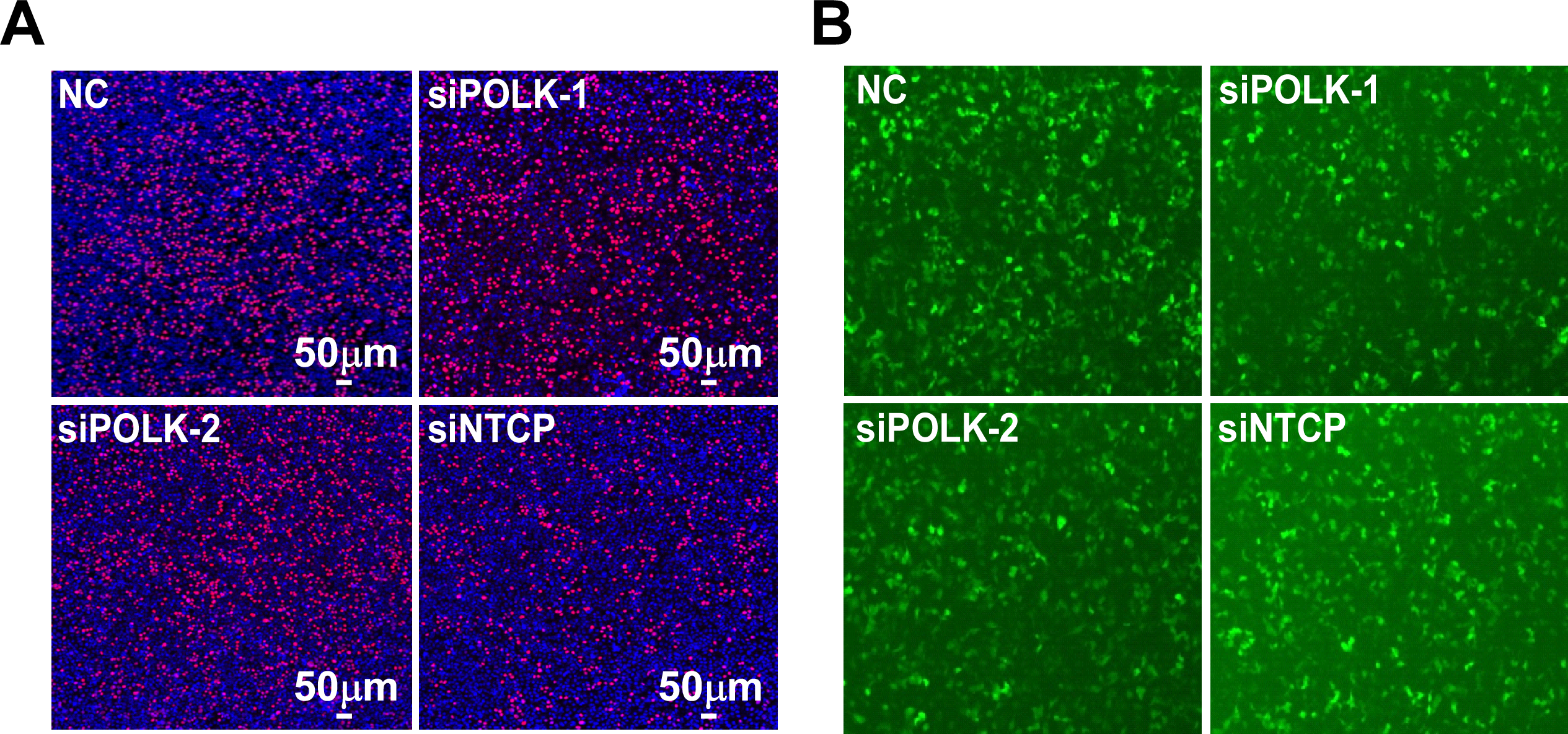

Supplement: S7 Fig — HepG2-NTCP cells were transfected with siRNA targeting NTCP or POLK or with a scramble siRNA as negative control (NC). Three days post siRNA transfection, the cells were inoculated with HDV for 24 h or lentivirus-VSV-GFP for 8 h. HDV infection was detected by immunostaining for HDV delta antigen with 4G5 antibody (red). Images were examined using a Nikon A1-R confocal microscopy; scale bars, 50μm (A). Lentivirus-VSV-GFP infection was recorded by fluorescence microscope on 3 dpi (B). (TIF) [file ppat.1005893.s007.tif]

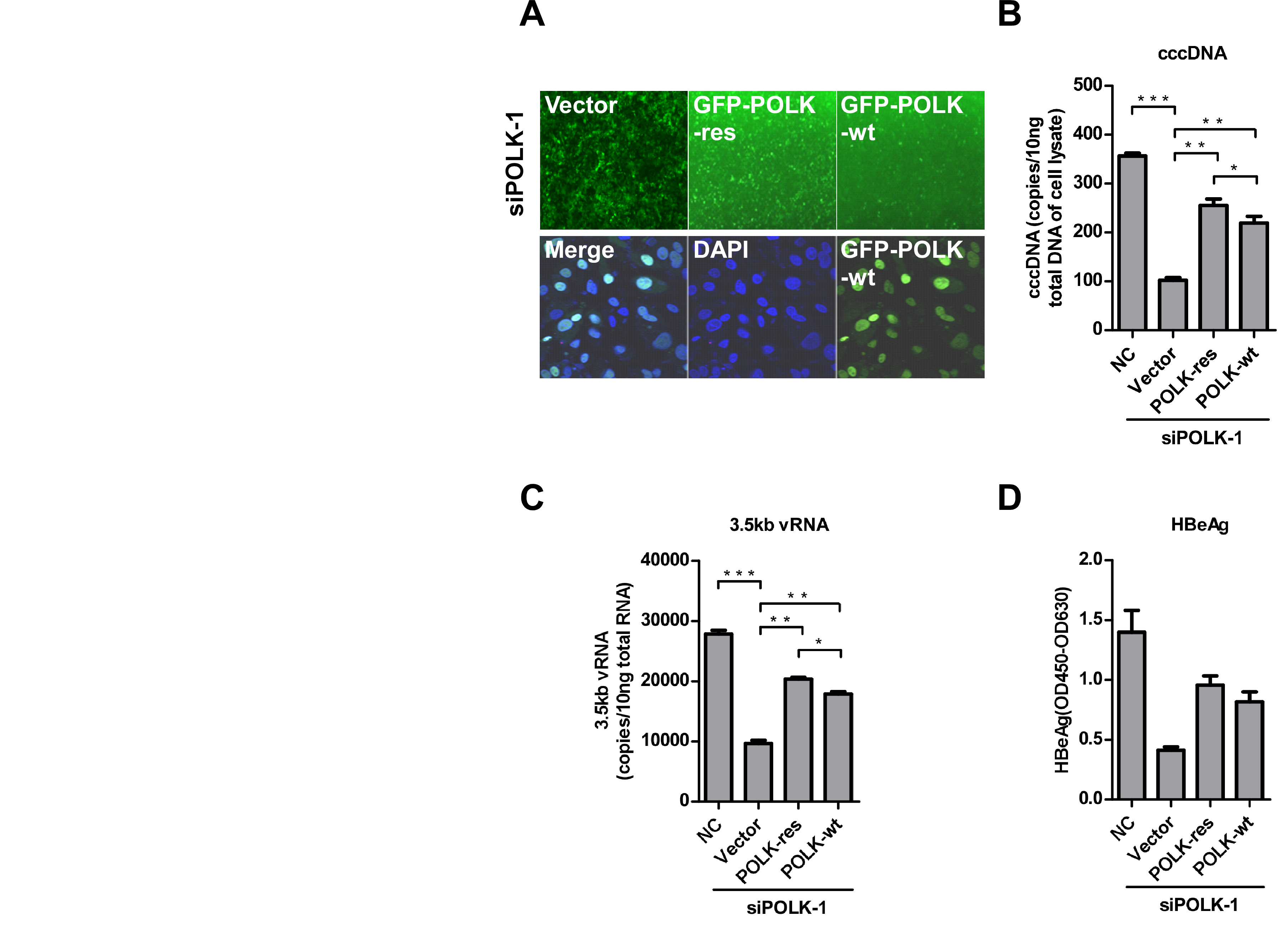

Supplement: S8 Fig — (A) HepG2-NTCP cells were infected with lentivirus encoding wild-type POLK (GFP-POLK-wt), or a siPOLK-1 resistant POLK (GFP-POLK-res), or GFP alone (Vector), respectively. Eight hours post lentiviral transduction, the cells were transfected with siPOLK-1. Ectopic expression of POLK was determined by fluorescence microscope 72 hrs after the transfection of siPOLK-1 (upper panel); The expression and localization of GFP-POLK-wt was analyzed by confocal microscopy (lower panel); nuclei were stained with DAPI (blue). (B-D) The HepG2-NTCP cells were infected with HBV 72 hrs after siRNA transfection. On 7 dpi, intracellular cccDNA (B) and 3.5kb vRNA (C) levels were quantified by qPCR assays. Secreted HBeAg was measured by ELISA (D). The data is representative of three independent experiments. Data were analyzed by an unpaired two-tailed t test. * p<0.05, ** p<0.01 and *** p<0.001. (TIF) [file ppat.1005893.s008.tif]

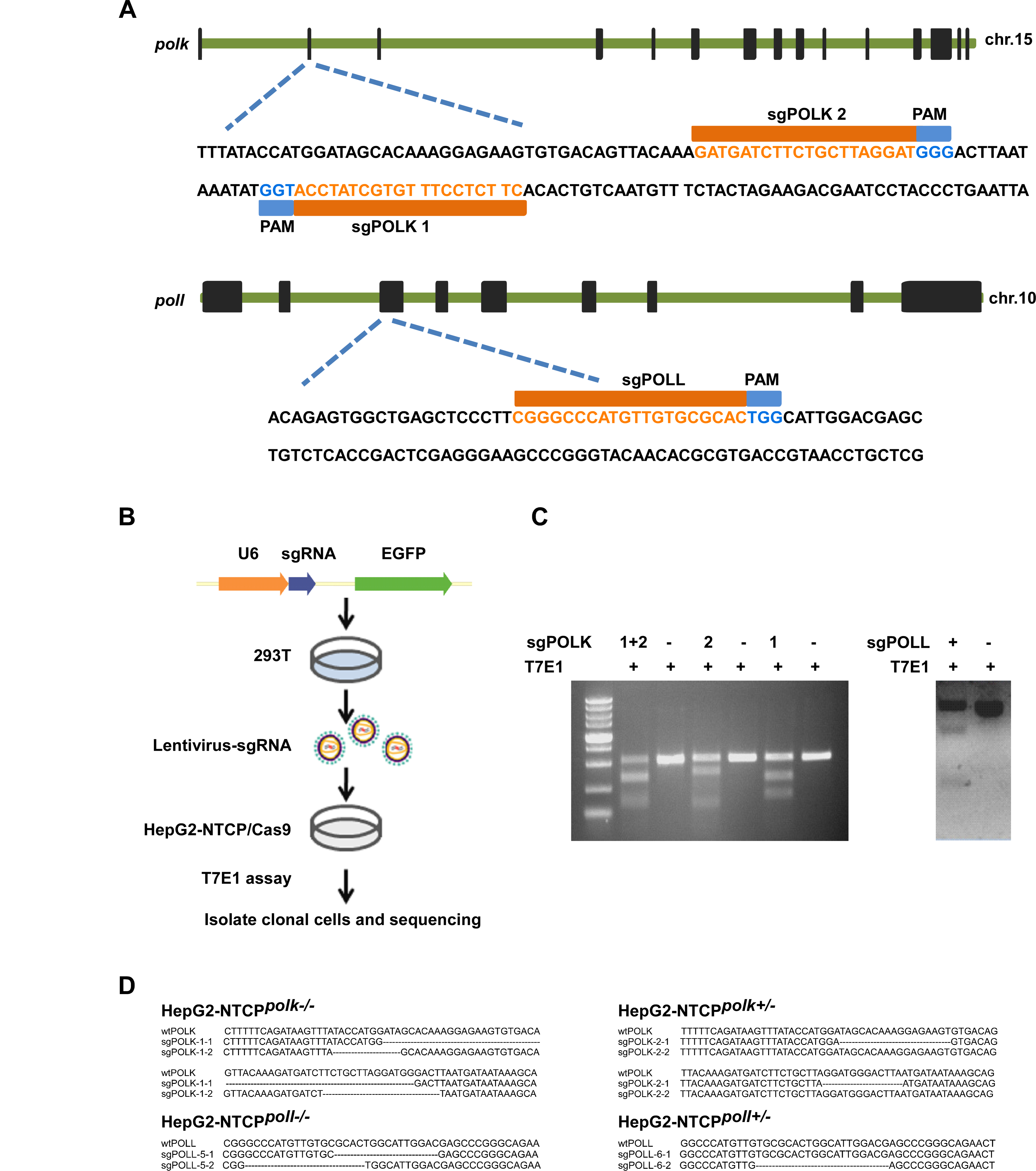

Supplement: S9 Fig — (A) Schematic representation of the single-guide (sg) RNA targeting sites at exon 2 of human polk gene (top) and at exon 3 of human poll gene (bottom). The sgRNA coding sequence is labeled in orange. The protospacer-adjacent motif (PAM) sequence is labeled in blue. (B) The procedure for generation of POLK, or POLL knockout HepG2-NTCP cells using CRISPR/Cas9 system is depicted. (C) DNA fragments containing sgRNA targeting region were amplified by PCR from genomic DNA of sgRNA transduced cells, the indel mutations were detected by T7E1 digestion of targeted PCR fragment. The left is for polk gene, the right is for poll gene. (D) Sequence alignment of wild-type polk gene (top), or poll gene (bottom) and the mutated alleles induced by sgRNA/Cas9 in isolated individual clonal cell lines, respectively. The wild-type sequence is shown at the top and deletions are shown as dashed lines. (TIF) [file ppat.1005893.s009.tif]

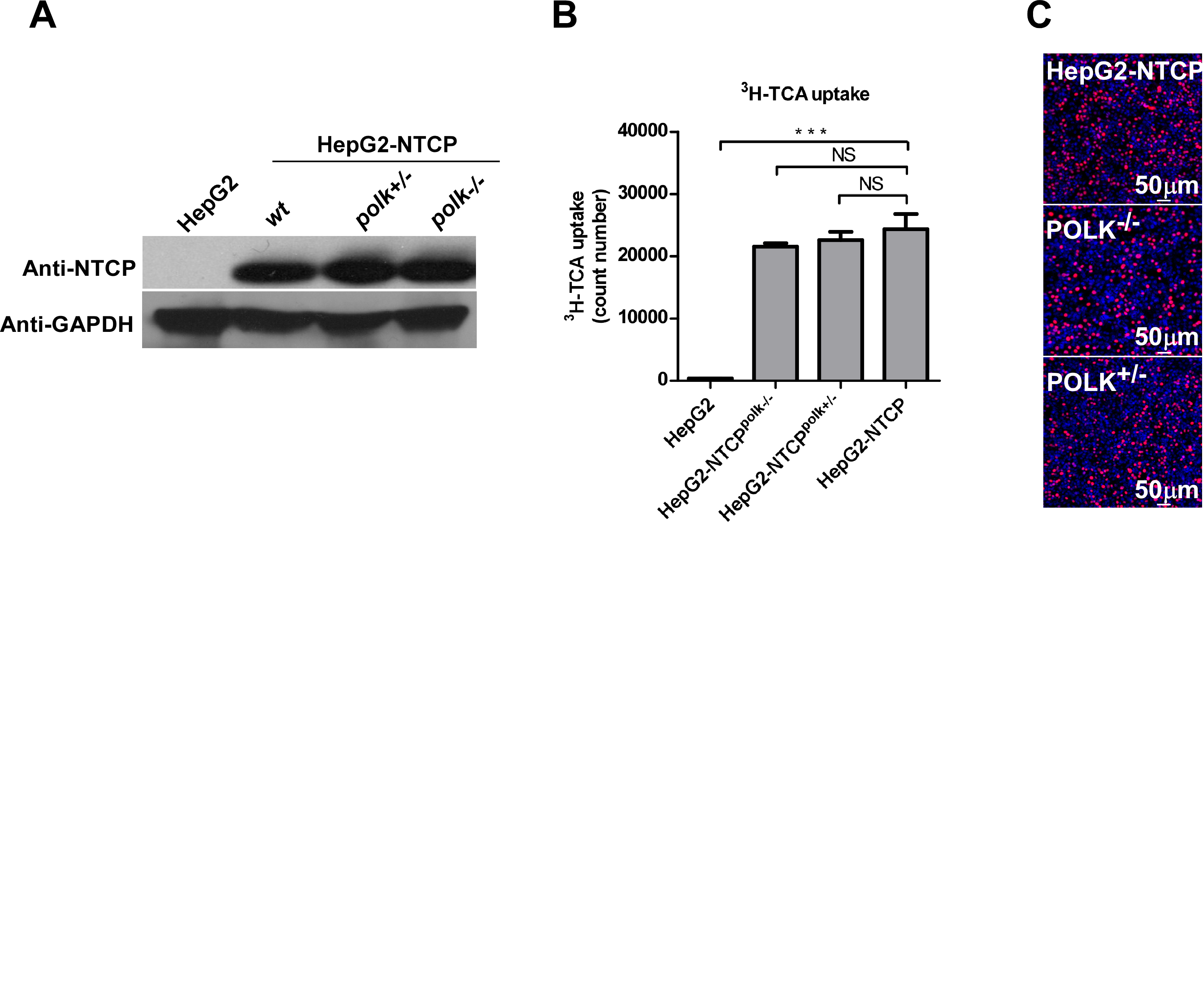

Supplement: S10 Fig — (A) Immunoblot analysis of NTCP expression in the indicated cell lines. GAPDH served as loading control. (B) [3H] taurocholate uptake efficiency was quantified by scintillation counting. HepG2 cell line was used as a negative control. Data are presented as Mean ± SD and representative of at least three independent replications. Data were analyzed by an unpaired two-tailed t test. NS: non-significant and *** p<0.001. (C) Parental HepG2-NTCP and the indicated HepG2-NTCP-derived cell lines were infected with HDV, the efficiency of HDV infection was determined by immunostaining for HDV delta antigen with 4G5 antibody (red) on 7 days post infection. Nuclei were stained with DAPI (blue). Images were examined using a Nikon A1-R confocal microscopy. Scale bars, 50μm. (TIF) [file ppat.1005893.s010.tif]
