# Supplementary material for: DNA Polymerase κ Is a Key Cellular Factor for the Formation of Covalently Closed Circular DNA of Hepatitis B Virus
Source: PLoS Pathog. 2016 Oct 26;12(10):e1005893. doi: 10.1371/journal.ppat.1005893 (PMC5081172; doi:10.1371/journal.ppat.1005893)
Supplement: S1 Table — (PDF) [file ppat.1005893.s011.pdf]

Supporting Information

S1 Table. Targeting sequences of siRNAs used in the study

siRNA ID	Target gene	siRNA sequence
NC	negative control	5'-UAAGGCUAUGAAGAGAUAC
siNTCP pool	sodium taurocholate cotransporting polypeptide	5'-GGAUCGUCCUCAAAUCCAA
		5'-GGAGUCAGCCGGAGAACAA
		5'-GGACAAGGUGCCCUAUAAA
		5'-GGUGCUAUGAGAAAUUCAA
siPOLA	polymerase (DNA directed), alpha	5'-GCACGCAAUAAAGACAAGA
siPOLB	polymerase (DNA directed), beta	5'-GCAGCAUCUGUUAUAGCAA
siPOLD1	polymerase (DNA directed), delta 1, catalytic subunit 125kDa	5'-CCCUCAAGGUACAAACAUU
siPOLD2	polymerase (DNA directed), delta 2, regulatory subunit 50kDa	5'-CCAUUGAUGGAGUCAGAUU
siPOLD4	polymerase (DNA-directed), delta 4	5'-CCUCAGGACAGAAGCGAGA
siPOLE	polymerase (DNA directed), epsilon	5'-CGGAAGCAGAUUUAAGGUG
siPOLG	polymerase (DNA directed), gamma	5'-GGUGCACAGACUUUAUGUA
siPOLH-1	polymerase (DNA directed), eta	5'-GCUCGUGCAUUUGGAGUCA
siPOLI	polymerase (DNA directed) iota	5'-GGAAAUUAUGAUGUGAUGA
siPOLK-1	polymerase (DNA directed) kappa	5'-CCAAUAGACAAGCUGUGAU
siPOLL-1	polymerase (DNA directed), lambda	5'-GGGAGAAGAAGCAGAAGAG
siPOLM	polymerase (DNA directed), mu	5'-GCGACACAUGUUGUGAUGG
siPOLN	polymerase (DNA directed) nu	5'-GCACCCAAUUCAGAUUACU
siPOLQ	polymerase (DNA directed), theta	5'-GGAAUGCCAUUUUCAAUUA
siPOLS	polymerase (DNA directed) sigma	5'-GGCUACGGUACCAAUAAUA
siPOLZ	polymerase (DNA directed) zeta	5'-AUGAGUAUGGAUCAUAUAC
siRev1	Rev1,polymerase (DNA directed)	5'-AUCGGUGGAAUCGGUUUGG
siPOLH-2	polymerase (DNA directed), eta	5'-CCAUUGGCUGUAGUAAGAA
siPOLK-2	polymerase (DNA directed) kappa	5'-AACCUCUAGAAAUGUCUCA
siPOLL-2	polymerase (DNA directed), lambda	5'-GCUGGACCAUAUCAGUGAG
tsNC	negative control for Tupaia hepatocytes	5'-UUCUCCGAACGUGUCACGU
sitsPOLK-1	polymerase (DNA directed) kappa, Tupaia	5'-GGAUAGGACUUAAUGACAA
sitsPOLK-2	polymerase (DNA directed) kappa, Tupaia	5'-GCAGCUAAGGAAAGCACAA
